# Supplementary material for: Perceptions of injury risk in the home and workplace in Nepal: a qualitative study
Source: BMJ Open. 2021 Mar 25;11(3):e044273. doi: 10.1136/bmjopen-2020-044273 (PMC7996655; doi:10.1136/bmjopen-2020-044273)
Supplement: Supplementary data [file bmjopen-2020-044273supp001.pdf]

BMJ Publishing Group Limited (BMJ) disclaims all liability and responsibility arising from any reliance placed on this supplemental material which has been supplied by the author(s)

Supplementary materials

Supplementary file 1. A topic guide for the focus groups

INTRODUCTION

- Firstly, thank participants for joining discussion group
- Give a brief introduction of yourself, the purpose of the study
- Explain approximate time (audio- recording, handwritten notes)
- Encourage participants to speak clearly and only one at a time
- Explain that there will be no right or wrong views but in line with the topic
- Assure anonymity and confidentiality of participants and their views
- Explain how participants will not be identifiable from any record or report arising from the discussion
- Explain participant does not have to talk about anything they feel uncomfortable with and can stop at any time for any reason
- Sign consent form (check that all participants have signed that they are happy for the FG to be audio-recorded)

Warm-up
Introduction by participants with a brief background.

FOR REFERENCE IF DEFINITION NEEDED:

Injury definition: "The physical damage that results when a human body is subjected to energy that exceeds the threshold of physiological tolerance or results in lack of one or more vital elements, such as oxygen". All types of injury will be included in this study, including those described as intentional or unintentional. The terms intentional and unintentional denote whether an injury was meant to harm the victim or not. Intentional injuries include suicide and self-harm, homicide, assault and child abuse or purposeful neglect. Unintentional injury is any injury that originated suddenly, without any intent of self-harm, homicide or suicide. For example, falls, road traffic collisions, accidental poisoning, fire/burns, animal related injuries (bite, sting, crush or attack).

Operational definition:
Home injury - Any injury defined above that occurs in the home environment but not related to work. (Work is any means by which someone earns money. So, if someone works from home, e.g., making something to sell, growing food / animals to sell – that is work and that would be an occupational injury). If you farm to grow food just for home/family use, that could be a home injury.

Occupational injury - Any injury defined above that occurs during paid employment at any location, including those who might be working without a wage but works for the benefit of their family (e.g. agriculture and animal husbandry).

DISCUSSION/INTERVIEW
Now we shall begin our discussion in an organized way. For this please think about the injury that occurs in the home environment/work environment to any age of people who live or work in your community. Please start with an example you know about. In addition, please give us some examples that you have seen in your home/working area.

[To organize the group discussion, use the following questions to guide the discussion]:

Part 1. Perceptions of injuries
1.1 What do we mean by injury?
1.2 What injuries have you experienced / witnessed in the home/work environment (as appropriate)

Part 2. Risk factors for injuries

Supplementary files

2.1 What caused those injuries in the home/work environment (as appropriate)? How did those injuries happen?

Part 3. Suggested intervention for injury prevention
3.1 What could be done (and by whom) to prevent other people having home/work injuries?
3.2 Home environment (as appropriate) - Whether the community people have had any change in their home environment and if so what that involved, and whether they think it helps? Also, do they have any safety equipment? If so, who provided it? Is it maintained? Does anyone check they are using it?
3.3 Work environment (as appropriate) - Whether the workers have had any safety training for their job and if so, what that involved and whether they think it helps? Also, do they have safety equipment? If so, who provides it? Is it maintained? Does anyone check they are using it?

Part 4. Barriers to and facilitators for such interventions
4.1 What might prevent such actions being done in the home/work environment (as appropriate)?
4.2 What might help such actions being done in the home/work environment (as appropriate)?

WRAP-UP

[At the end, there will be a short session for summarizing on a flip chart and recapping the whole discussion, thanking the participants and serving refreshments. This will be followed immediately by a meeting between the facilitator and note-taker]

Ending the discussion by saying: We have been discussing for about an hour. Do you think we missed anything that needs to be discussed?

FG discussion recording details

**Date:**
**Palika's name:**
**Ward No.:**
**Location (venue):**
**Type of Target Group:**
**Number of participants:**
**Age group:**
**Level of education:**
**Length of session:**
**Facilitator's name**
**Note taker' name:**
**Observation (if any):**
**Recorder No.:**

Supplementary files

Supplementary file 2. A semi-structured interview schedule for key interviews

INTRODUCTION

Explain the study, ask if the participant has read the information sheet. Use the information sheet to explain the purpose of study, the voluntary nature of the interview, that the information they give will be kept confidential and that they can withdraw at any time.

Audio-recording: explain and ask for consent to use.

Consent: explain and ensure consent form is signed has a thumb print.

FOR REFERENCE IF DEFINITION NEEDED:

Injury definition: "The physical damage that results when a human body is subjected to energy that exceeds the threshold of physiological tolerance or results in lack of one or more vital elements, such as oxygen". All types of injury will be included in this study, including those described as intentional or unintentional. The terms intentional and unintentional denote whether an injury was meant to harm the victim or not. Intentional injuries include suicide and self-harm, homicide, assault and child abuse or purposeful neglect. Unintentional injury is any injury that originated suddenly, without any intent of self-harm, homicide or suicide. For example, falls, road traffic collisions, accidental poisoning, fire/burns, animal related injuries (bite, sting, crush or attack).

Operational definition:

Home injury: Any injury defined above that occur in the home environment (home and near environment) but not related to work. (Work is any means by which someone earns money. So, if someone works from home, e.g., making something to sell, growing food / animals to sell – that is work and that would be an occupational injury). If you farm to grow food just for home/family use, that could be a home injury.

Occupational injury: Any injury defined above that occurs during work at any location, including those who might be working without a wage but works for the benefit of their family (e.g. agriculture and animal husbandry).

Opening:

We would like to ask you a few questions about injuries, how they happen and what might prevent them. There are no right or wrong answers; we just want to find out what you think helps to keep people safe from accidents and what makes it hard to do this.

1. What is your understanding of what an injury is? Can you give an example, please?
   (*if they are not correct, explain what we mean by the word 'injury')*

2. What injuries have you experienced / witnessed in the home/work environment (as appropriate)? Can you describe one instance?

   (*If they can't give an example, ask them to imagine a near miss or something that they could envisage happening.)*

3. What do you think might have caused those injuries/why do you think they happened? How did those injuries happen? Can you describe the reasons, please?

   (Try and get them to give as much detail as possible. Repeat what they have said and ask them more details, if need be.)

4. Do you have any ideas about how injuries in the home/at work (as appropriate) could be prevented?
   a. What sort of thing might help?
   b. Who might do this?
   c. How might this be done?

Supplementary files

5. Have you made any changes to your home/work to try and reduce the likelihood of an injury happening? Have you tried to make some changes?

   a. Has anyone in the community (home or work) made any changes? If yes, what? If yes, do you think it helped?
   b. Is any safety equipment used in the home/work at all? If yes, what and who provided it? Is it maintained (if relevant)? Does anyone check it (if relevant)?

   (They might not be in a position to make any changes so ask whether they have ideas for what could have been done and if they have made suggestions to anyone.)

6. **Work environment:** have the workers had any safety training for their job, this includes any training for use of safety equipment? Who provided it? Is this undertaken regularly? Who ensures that it is completed?

Barriers and facilitators to injury prevention

7. What might prevent or deter people trying to use an intervention to stop injuries (home or work)? Why might people not use something that will help to prevent an injury? Do you have any ideas?

   (Try to get some detail here about cost, practicality, someone taking ownership, leading the intervention etc.)

8. What might help or encourage people to use an intervention to stop injuries (home or work)? Do you have any ideas?

   (Try to get some detail here about cost, practicality, someone taking ownership, leading the intervention etc.)

9. Do you have any other comments or anything else that you would like to say?

Thank you very much for talking to me. We will use the information that you have given me to describe the hazards that cause injuries and the possible interventions that might be used to help prevent them.

KIIs recording details

**Date:**
**Palika's name:**
**Ward No.:**
**Location (venue):**
**Type of Target Group:**
**Age group:**
**Level of education:**
**Length of interview:**
**Interviewer's name:**
**Observation (if any):**
**Recorder No.:**
**Personal identification code**

Supplementary files
