# Supplementary material for: Perceptions of injury risk in the home and workplace in Nepal: a qualitative study
Source: BMJ Open. 2021 Mar 25;11(3):e044273. doi: 10.1136/bmjopen-2020-044273 (PMC7996655; doi:10.1136/bmjopen-2020-044273)
Supplement: Author's manuscript [file bmjopen-2020-044273.draft_revisions.pdf]

BMJ Open is committed to open peer review. As part of this commitment we make the peer review history of every article we publish publicly available.

When an article is published we post the peer reviewers' comments and the authors' responses online. We also post the versions of the paper that were used during peer review. These are the versions that the peer review comments apply to.

The versions of the paper that follow are the versions that were submitted during the peer review process. They are not the versions of record or the final published versions. They should not be cited or distributed as the published version of this manuscript.

BMJ Open is an open access journal and the full, final, typeset and author-corrected version of record of the manuscript is available on our site with no access controls, subscription charges or pay-per-view fees (http://bmjopen.bmj.com).

If you have any questions on BMJ Open's open peer review process please email info.bmjopen@bmj.com

Barriers and enablers for injury prevention at home and workplace in Nepal: a qualitative study

Journal:	*BMJ Open*
Manuscript ID	bmjopen-2020-044273
Article Type:	Original research
Date Submitted by the Author:	28-Aug-2020

Complete List of Authors:	Joshi, Elisha; Kathmandu Medical College and Teaching Hospital, Community Medicine<br>Bhatta, Santosh ; University of the West of England, Faculty of Health and Applied Sciences<br>Deave, Toity; University of the West of England, Bristol, Faculty of Health and Applied Sciences<br>Mytton, Julie; University West of England, Faculty of Health and Applied Sciences<br>Adhikari, Dhruba; Mother and Infant Research Activities<br>Manandhar, Sunil; Mother and Infant Research Activities<br>Joshi, Sunil; Kathmandu Medical College and Teaching Hospital, Community Medicine

Keywords:	QUALITATIVE RESEARCH, PUBLIC HEALTH, Health & safety < HEALTH SERVICES ADMINISTRATION & MANAGEMENT, PREVENTIVE MEDICINE, SOCIAL MEDICINE

SCHOLARONE™ Manuscripts

I, the Submitting Author has the right to grant and does grant on behalf of all authors of the Work (as defined in the below author licence), an exclusive licence and/or a non-exclusive licence for contributions from authors who are: i) UK Crown employees; ii) where BMJ has agreed a CC-BY licence shall apply, and/or iii) in accordance with the terms applicable for US Federal Government officers or employees acting as part of their official duties; on a worldwide, perpetual, irrevocable, royalty-free basis to BMJ Publishing Group Ltd ("BMJ") its licensees and where the relevant Journal is co-owned by BMJ to the co-owners of the Journal, to publish the Work in this journal and any other BMJ products and to exploit all rights, as set out in our licence.

The Submitting Author accepts and understands that any supply made under these terms is made by BMJ to the Submitting Author unless you are acting as an employee on behalf of your employer or a postgraduate student of an affiliated institution which is paying any applicable article publishing charge ("APC") for Open Access articles. Where the Submitting Author wishes to make the Work available on an Open Access basis (and intends to pay the relevant APC), the terms of reuse of such Open Access shall be governed by a Creative Commons licence – details of these licences and which Creative Commons licence will apply to this Work are set out in our licence referred to above.

Other than as permitted in any relevant BMJ Author's Self Archiving Policies, I confirm this Work has not been accepted for publication elsewhere, is not being considered for publication elsewhere and does not duplicate material already published. I confirm all authors consent to publication of this Work and authorise the granting of this licence.

For peer review only

Barriers and enablers for injury prevention at home and workplace in Nepal: a qualitative study

Corresponding Author

1. Elisha Joshi*

Nepal Injury Research Centre, Kathmandu Medical College, Duwakot, Bhaktapur, Nepal
ejoshi03@gmail.com; https://orcid.org/0000-0002-3534-276X

Co-Authors

2. Santosh Bhatta*

Faculty of Health and Applied Sciences, University of the West of England, Bristol, UK
https://orcid.org/0000-0001-6393-2495

**Elisha Joshi and Santosh Bhatta have contributed equally*

3. Toity Deave

Faculty of Health and Applied Sciences, University of the West of England, Bristol, UK
https://orcid.org/0000-0001-9966-8948

4. Julie Mytton

Faculty of Health and Applied Sciences, University of the West of England, Bristol, UK
http://orcid.org/0000-0002-0306-4750

5. Dhruba Adhikari

Mother and Infant Research Activities (MIRA), Makwanpur, Nepal

6. Sunil Manandhar

Kathmandu Medical College and Mother and Infant Research Activities (MIRA), Makwanpur, Nepal
https://orcid.org/0000-0002-6583-3973

7. Sunil Kumar Joshi

Department of Community Medicine, Kathmandu Medical College Public Limited, Kathmandu, Nepal
https://orcid.org/0000-0002-2704-5060

Word Count: 3888 (Max 4000 words)

For peer review only - http://bmjopen.bmj.com/site/about/guidelines.xhtml

For peer review only

ABSTRACT

Objective: Injuries are a global health problem. To develop context specific injury prevention interventions, one needs to understand population perceptions of home and workplace injuries. This study explored a range of views on home and workplace injuries and identified barriers and facilitators to injury prevention.

Design: Qualitative study: interviews and focus groups.

Setting: Three municipalities: Hetauda sub-metropolitan city, Thaha municipality (urban) and Bakaiya rural municipality in the Makwanpur, Nepal.

Participants: Nine focus groups (74 participants) and nine interviews were completed; workers from diverse occupations, residents (slum, traditional or modern homes), health workers and local government decision makers participated in the study between May and August 2019. The interviews and discussions were audio-recorded, transcribed verbatim, translated into English and analysed thematically.

Results: Five themes were developed: unsafe home and workplace environment; inadequate supervision and monitoring; safety takes low priority- financial and behavioural considerations; safety education and training; government led safety programmes and enforcement. The key barrier to injury prevention was perceived to be lack of knowledge about injury risk and preventive measures at both a community level and in the workplace. The facilitators were community level educational programmes and health and safety training to employees and employers. Participants stressed the importance of the role of government in planning future injury prevention programmes in different environments.

Conclusions: This study highlighted that both home and workplace injuries are complex and multifactorial. Most importantly, lack of knowledge about injury risks and preventive measures, at both a community level and in the workplace, were found to be common barriers to injury prevention; these were perceived to be mitigated by educational programmes. Together with previously published epidemiological evidence, the barriers and facilitators identified in this study provide a useful basis to inform policy and practice.

Keywords: Barriers and facilitators, injury prevention, home, workplace, qualitative, Nepal

Strengths and limitations of this study

- We included a diversity of participants who lived in different home environments, from different work settings and from different socio-economic backgrounds.
- We believe this is the first ever study that has explored, qualitatively, the range of views of participants about home and workplace injuries in Nepal.
- The study is not able to provide perceptions about injury risks and preventive measures by injury type.

For peer review only

INTRODUCTION

Injuries are a global health problem accounting for more than five million deaths each year.(1) Ninety-percent of injury-related deaths occur in low-and middle-income countries (LMICs) and, in Nepal, there has been an increase in injury-related deaths from 6.3% to 9.2% between 1990 and 2017.(2) Globally, road traffic injuries, falls, burns, poisoning and suicide are the leading causes for unintentional and intentional injuries.(3) According to the International Labour Organization more than 2.78 million deaths per year are estimated to be due to occupational injuries or workplace disease.(4) In Nepal, 200 workers die and 20,000 workers suffer from workplace injuries yearly.(5)

With the increase of qualitative literature on injuries occurring at home,(6) parental supervision and teaching children about injury risks were considered facilitators, while barriers to child injury prevention were identified as parents' lack of anticipation of injury risks and perceiving injuries as inevitable events.(7) Culturally acceptable prevention measures, appropriate supervision arrangements, separation of hazards and training children and parents about safety were suggested by a study of community perceptions in Makwanpur, Nepal.(8) Two community-based studies conducted in rural Nepal emphasised that unintentional child injuries were thought to be due to coincidence, bad luck, witchcraft or ill fate.(8, 9) Likewise, in Bangladesh, child drowning was believed to be a result of ill fate and unpreventable.(10) Rarely were the environmental and infrastructural factors thought to be the cause of child injury by parents.(8) While conflicts with husband, illiteracy, financial burden were found to increase suicidal risk for Nepalese women.(11)

Workplace injuries are becoming a public health concern in all LMICs. One qualitative study undertaken in Bangladesh found that poor people were at greater risk of injury, employers were reluctant to take responsibility for workers and subcontracting workers was observed to increase the injury risk.(12)Despite the high level of awareness about the use of personal protective equipment (PPE) among Nepali workers, there was low practice of using PPE.(13) A qualitative study conducted among Nepali migrant workers suggested that workplace injuries were due to lack of health and safety regulations, the risk-taking behaviour of workers and perceived work pressure.(14)

Human behaviour, being a complex phenomenon, is determined by various factors like environmental factors (such as social support/barriers, ability to change one\s own environment), behavioural factors (such as skills, practice and self-efficacy) and personal factors (such as knowledge, perception).(15) To understand health and safety behaviours, one needs to understand how people perceive the risks and what the factors are that influence their behaviours.(13) Little is known about how the people of Nepal perceive and deal with home and workplace injuries, its risk

For peer review only - http://bmjopen.bmj.com/site/about/guidelines.xhtml

factors and preventive measures during their daily life activities. This study explored a range of views on home and workplace injuries and identified barriers and facilitators to injury prevention.

METHODS

Study design

We adopted a qualitative research methodology using focus group discussions and key informant interviews.

Study participants and setting

We sampled, purposively, the residents and people working in different sectors and three topographical areas of Makwanpur district, Nepal, to capture the diverse views of the people. This included Hetauda sub-metropolitan city (urban area), Thaha municipality (suburban area) and Bakaiya municipality (rural area). Members of the research team consulted with municipality officers to identify and invite the appropriate individuals and groups of participants. The inclusion criteria were 18 years old or over and resident and/or working in one of the three topographical areas. The aim was to ensure diversity of location (rural, urban), occupation, housing type and key government personnel. Identified individuals were invited to take part, were given a participant information sheet and consent form. Consent forms were completed for those who agreed to take part. The research team conducted all the focus groups and interviews at a location and time convenient to the participant groups and individuals in each study site.

Data collection

A topic guide for focus groups and semi-structured interview schedule were developed. These were developed based on previous work undertaken by the research team, pretested and finalised prior to data collection. All focus groups and interviews were conducted face-to-face by either SB or EJ. With consent, the focus groups and interviews were digitally audio-recorded and written notes were taken concurrently by a note taker to capture non-verbal communication. The interview schedule and topic guide asked questions about, for example, perception about injuries, risk factors and barriers and facilitators to injury prevention.

Data management

Recordings were transcribed verbatim in Nepali and then translated into English with an aim to retain the original meaning of the statements. The transcriptions and translations were completed by experienced Mother and Infant Research Activities (MIRA) staff and verified by researchers (EJ &

For peer review only - http://bmjopen.bmj.com/site/about/guidelines.xhtml

SB). Personal identification information for participants was removed from transcriptions and replaced with a unique identification code.

Data analysis

The interviews and focus groups data were analysed using inductive thematic analysis to search for repeated patterns of meaning or themes in the data.(16) NVivo qualitative data analysis software, Version 12 was used to arrange the codes systematically and collate data relevant to each code.(17) Two transcripts were coded by both EJ and SB and discussed with a third researcher (TD) to agree an initial coding framework which was then applied consistently across the transcripts. After coding all transcripts, the individual codes were reviewed and placed into clusters based on their similarities avoiding duplication. The clusters were organised to develop candidate themes. Any discrepancies and difference in codes and themes developed were discussed with a third researcher (TD) and final themes were agreed.

RESULTS

Participants' characteristics

We conducted nine focus groups and nine interviews with a total of 83 participants across three municipalities. One participant refused to participate in the interview. The average length of each focus group was 37 minutes and interview was 31 minutes. A total of six focus groups and six interviews were conducted in Hetauda sub-metropolitan city, two focus groups and two interviews in Bakaiya rural municipality and one focus groups and one interview in Thaha municipality. Participants in the focus groups were from similar backgrounds (table 1) whereas interviewees were representatives of different institutes and organisations (table 2).

For peer review only

Table 1. Characteristics of the focus group participants

Type of Injury	Participants	Participant numbers	Age range of participants (years)	Gender	Locations
Home (FG 1)	Residents in rural, traditionally built home	8	21-55	M:4 F:4	Rural Area
Home (FG 2)	Residents in rural, concrete/modern home	9	21-55	M:5 F:4	Rural Area
Home (FG 3)	Residents in urban area	8	35-50	M:4 F:4	Urban Area
Occupational (FG 4)	Haulage/truck drivers	9	40-57	M:9	Urban Area
Home (FG 5)	Residents in slum area	8	18-59	M:4 F:4	Urban Area
Occupational (FG 6)	Skilled construction workers: plumber, welder, electrician	8	21-50	M:8	Urban Area

For peer review only - http://bmjopen.bmj.com/site/about/guidelines.xhtml

Occupational (FG 7)	Farmers (agriculture, animal husbandry)	8	37-51	M:4 F:4	Rural Area
Occupational (FG 8)	Factory workers (cement factory)	9	26-41	M:8 F:1	Urban Area
Occupational (FG 9)	Trade professionals	7	30-53	M:7	Urban Area

Male: M, Female: F; FG: Focus Group

Table 2. Characteristics of the interview participants

Type of Injury	Participants	Age range of participants (years)	Gender	Locations
Home (KII 1)	President of mothers' group	40-50	F	Rural Area
Home & occupational (KII 2)	Consultant in charge of hospital emergency department	20-30	F	Urban Area
Home & occupational (KII 3)	Healthcare Manager of municipality	40-50	M	Rural Area
Home & occupational (KII 4)	Healthcare Manager of sub-metropolitan city	40-50	F	Urban Area
Occupational (KII 5)	Trade union representatives	50-60	M	Urban Area
Occupational (KII 6)	Senior Officer of Hetauda industrial area	40-50	M	Urban Area
Occupational (KII 7)	Representative of transport workers	40-50	M	Urban Area
Home & occupational (KII 8)	Rural municipality officer	40-50	M	Rural Area
Home (KII 9)	Senior executive of education	50-60	M	Urban Area

KII: Key Informant Interview

Themes

Five themes were identified from the focus groups and interview data and these are described below. Relevant quotes are presented to illustrate the key themes.

1. Unsafe home and workplace environment

Physical infrastructure and home environment were perceived as common causes of unintentional and intentional injuries at home. The participants said that the lack of railings on stairs was the leading cause of fall injuries among people living in traditionally built rural houses, especially hazardous to children. Participants from urban areas raised their concerns that elderly people sustained fall injuries due to slipping on marble or tiled bathroom floors. Burn injuries were common among children and women where firewood was used for cooking.

Along with the infrastructural aspect, most of the participants also stressed that family dynamics led to intentional injuries. For example, poverty, illiteracy and drinking alcohol were perceived as an underlying root cause of disputes in the family; the most common disputes took place between husband and wife. Participants were concerned that the recent change in social structure, where

For peer review only

For peer review only - http://bmjopen.bmj.com/site/about/guidelines.xhtml

many men sought work outside Nepal. This was reported to have led to an increase in self-harm among wives of these men to save themselves from social shame as a result of their extra marital affairs with other men. Children of these families are likely to be vulnerable to abuse because of the lack of appropriate parenting.

> *The females might feel that she has to cut the grass and even take care of the kids herself while the males are just busy drinking alcohol. So, this cause a dispute between them [husband and wife] and with this they might get injured (laughs). Some die by eating poison …* (FG 1, P8)

Regarding workplace injuries, different professionals, like factory workers, healthcare staff and farmers all highlighted that falls, cuts and burns were common injuries. Some described how they have to work in risky environments sometimes without safety equipment.

> *I have seen workers suffering from the pain due to heat. They have to take out rods from 300 degrees of heat by bare hands. They even don't wear gloves and boots. They use scissors [instead of tongs] ….* (KII 6)

Many participants explained that workers who are illiterate and suffer from poverty were those who worked under private contractors.

2. Inadequate supervision and monitoring

The group discussions highlighted the need for close supervision and monitoring of children and teenagers. Although parents said they were aware of this, they said that careful supervision was difficult for working and single parents and, therefore, injuries occurred.

> ***…****Two kids died by drinking milk in which the sugar and medicine [Insecticide] to kill the flies was mixed. …. They were twins of only 5 years of age. While the others went to the field to collect potatoes, the kids drank all the milk …* (FG 2, P9)

Most participants were concerned about the rise in teenagers threatening to, and actually, self-harming. They believed that teenagers acted like this to pressurise their parents to fulfil their demands.

> *We can't even say a word to the kids these days. My child is only 10-11 years old and he listens to a lot of things from outside. If I scold him a bit, he says he will eat poison and whatever. When I scold him again for saying that, he tries to threaten me. I think it's all due to this internet nowadays.* (FG 2, P2)

For peer review only - http://bmjopen.bmj.com/site/about/guidelines.xhtml

A participant who had survived an attempted suicide explained that parents need to talk and guide their children to try to avoid such circumstances.

3. Safety takes low priority: financial and behavioural considerations

Most participants said that when they were at home they didn't think about safety. Carelessness when completing tasks in a hurry was the most common reason for injuries occurring at home and workplace.

> *Most of the injuries take place due to our carelessness. Like we throw nails and other sharp materials here and there…. Another reason is … performing any kind of activities hurriedly while walking …* (FG 2, P2)

Many participants felt that injuries occurred by chance, accidentally or suddenly and that they were part of normal day-day life. They believed that such injuries occurring abruptly could not be prevented, despite them being careful while performing any task.

> *I feel that people get injured if they have misfortune or if the movement of planets that influences the destinies of people is not good or else, they [people] won't get injured. Even if we are careful, we might get injured due to our bad fortune.* (FG 5, P2)

Participants from rural villages confessed that financial constraints hindered them from implementing safety measures like installing railings to balconies as they struggled to meet their basic needs.

> *Due to this economic barrier, we are unable to manage everything…* (FG 1, P5)

Participants described the common practice of employers who pressurised their workers to work fast without providing personal protective equipment (PPE). Inadequate capital was one reason for employers for not providing PPE or installing safer, automatic machines.

> *…the manufacturers of construction factories like grill factories are more concerned about how to increase their production leading the workers to work fast and carelessness at work.* (KII 5)

The workers stated that, not only did they have to work without PPE, especially the contracted workers, but most of them worked carelessly, misusing or not using safety equipment even when the PPE was available. Overconfidence and feeling that nothing would happen to them led the workers take such risks.

> *Even if we have safety equipment, we get careless and do not use them thinking that the work we are about to perform just takes 2 minutes of time and we won't require it [safety equipment]. But, in that 2 minutes of time any incident may occur.* (FG 7, P3)

For peer review only - http://bmjopen.bmj.com/site/about/guidelines.xhtml

Senior personnel emphasized that the word supervisors should consider safety, raise awareness and orient the workers about the dangerous aspects of their work.

> *What I would say is once you recruit the person, he/she should be taught everything. Every industry should manage this and conduct orientation. The industry should make employees learn about the culture of work. But this culture isn't practised.* (KII 6)

4. Safety Education and Training

Additional to the behavioural and financial aspects, participants reported that lack of awareness was a major contributing factor that led to home and workplace injury; illiteracy and a lack of understanding about injury was prevalent. Almost all the participants voiced the need for an awareness programme, that it would be a crucial enabling factor for injury prevention, both in the home and workplace.

> *In case of home, there should be awareness program because they [community people] don't know. They should be educated such that they can bring change in their behaviour. It is because they don't understand.* (KII 3)

Regarding home injury, many participants stated that a programme to reduce injuries at home should be conducted by local community groups and their leaders, such as the mothers' groups. Participants believed that effectiveness of programmes relied on being led by injury experts in collaboration with Non- Governmental Organizations (NGOs), International Non- Governmental Organisations (INGOs) and local government.

> *If the outsiders go and tell them [community people] they might listen. But if we locals tell them they won't listen to us.* (FG2, P4)

Participants also felt that such injury prevention programmes should be delivered to school children, teachers, parents and community leaders, using social media or community mobilisation.

> *For that we must gather all the family members in a place and discuss about this matter [injuries]. We must discuss about how injuries take place and how we can reduce it [injuries]....* (FG 3, P7)

Factory workers raised concerns about the lack of institute to train workers, to make them aware of occupational health and safety. Skilled workers and laborers highlighted the need for safety training, that it could be provided and monitored by industries, NGOs and the Ministry of Labour. There was a consensus among factory workers that training needed to be inclusive of all workers, company and contract staff, including the employers, that it should be done regularly, addressing any technological

For peer review only - http://bmjopen.bmj.com/site/about/guidelines.xhtml

updates and human tendency to forget. Pamphlets or brochures with brief messages would raise awareness amongst the workers.

> *The main important thing is to [make aware] the employer, those who employ the workers. Basically, they should be aware of the health of the workers. We should also be able to make the workers understand that, if you are healthy then you can do lot of work...* (KII 5)

5. Government led safety programmes and enforcement

Almost all participants thought that the government's role was pivotal for injury prevention activities. They thought that government lacked focus on injury prevention activities due to the lack of injury data. Most participants believed that the local government authorities needed to take a lead on injury prevention activities. On probing what would work, they thought that collaboration with NGOs, INGOs, and injury experts strategic planning could be achieved. Participants provided examples of how the municipality was working in partnership with some organisations for community health development and women empowerment. For example, the municipality should work with research organisations to prioritise injury prevention programmes.

> *The local government along with the NGO/INGOs have mobilized the mother's group... The local government should launch such a program [injury prevention] which will enhance them [mothers] and help to promote the injury related activities.* (KII 9)

The participants highlighted the role of central government could be to formulate uniform policies, with strict enforcement of rules and regulations to prevent injuries both in the home and workplace. Periodic monitoring visits to industries undertaken by the government and private sectors to ensure safety standards were being followed was felt to be mandatory.

> *The government of Nepal should formulate standard protocol to prevent injuries at industries. If there isn't environment as mentioned in the protocol then they shouldn't be renewed and the industry should be closed immediately.* (KII 3)

Barriers and facilitators

The key barrier to injury prevention identified by participants was the lack of knowledge about injury risk and preventive measures, at both a community level and in the workplace. Other barriers included: safety not being a priority, inability to improve the safety of home or working environments due to financial constraints. The barriers, particularly for child injury prevention, included the inability of parents to provide adequate supervision. Facilitators for injury prevention were three-fold. At a community level, there could be provision for an educational programme to

For peer review only - http://bmjopen.bmj.com/site/about/guidelines.xhtml
For peer review only

raise injury prevention awareness. To enable workplace injury prevention measures, health and safety training could be provided for employers and employees, introduction of safety laws and their rigorous enforcement. The involvement of external agencies and involvement of government authorities could facilitate the implementation and effectiveness of these programmes and training.

DISCUSSION

This study found that injuries were perceived as a problematic issue and preventive measures have been neglected

Home injuries

In our study, the home environment and socioeconomic aspects were highlighted as the main contributing factors for injuries such as falls, burns and poisonings. Unsafe buildings and cooking on open fires have been identified previously as injury hazards(18) and, in a countrywide population-based study conducted in Nepal, falls were found to be the leading cause of injury.(19) A household survey that assessed home hazards for child injury in rural Nepal, found that 98% households did not have protective railings on stairs, more than 80% households had no window bars and 50% households lacked a protective barrier for their balconies.(20) Our study participants blamed their financial constraints for being unable to install such protective equipment.

The findings from our study supports those from previous epidemiological evidence where children were found to be at high risk of burns and that the home was hazardous.(21, 22) Participants in our study provided the context and reported reasons for some of the more common injuries identified by other studies; for example, open fires for cooking is unsafe(23) and the majority of households (61%) in rural areas of Nepal had chemicals or fertilisers within the reach of children.(20)

Some parents believed that injuries were a normal part of child development, bad luck, witchcraft or ill fate. This fatalism, in relation to injury, has been reported in previous studies conducted in LMICs(6, 24, 25) but no method to address this belief has been developed. A qualitative study in Nepal showed that when people are unable to rationalize the cause then the concept of fate is ascribed.(26) This lack of knowledge in the community has been reported in previous studies conducted in Nepal.(8, 9) In line with previous studies, the participants of this study also highlighted the need for interventions at all levels, such as government, NGOs and the local community.(27)

For peer review only

Workplace injuries

Minor injuries were reported as being common occurrences within the workplace but fatal injuries and those that led to long-term disability were also reported. Just like for home injuries, study participants believed that poverty and illiteracy were interlinked and were the root cause of workplace injuries. This might be because employers tend to hire the poorer workers who are ready to take on risky jobs but with a relatively higher wage(12) or that, similar to our study, carelessness was a significant causal factor, as found in one study of Iranian workers.(28)

Study participants stressed the need to raise awareness amongst workers about the importance of personal protective equipment (PPE) use via regular and periodic occupational health and safety education programmes. A study in India demonstrated that the understanding of occupation hazards was significantly associated with the literacy status of workers and delivering occupational health education programmes augmented the understanding of Occupational Health and Safety (OHS).(29) Another study suggested that such programmes should be delivered together with behaviour change counselling(30) but our study participants believed that awareness programmes, with adequate PPE and enforcement of occupational standards, would be suffice for adopting safe workplace practices. One study found that high levels of awareness of occupational hazards and use of PPE did not lead to the actual use of PPE.(31) Further exploration is needed about why workers don't use PPE despite the availability and training. Unlike other studies, which highlighted lack of user-friendly PPE and ineffective PPE(32, 33), our study found an absence of formal training prior to commencing work and lack of PPE which created environments conducive to injuries.

In Nepal, the 1992 Occupational Health and Safety law did not specify how to evaluate or enforce the law.(34) After the revision in 2017, it explicitly describes the roles of employees and employers in adhering to OHS standards, yet legislation is silent on the supervision and monitoring of the implementation status.(35) In line with other studies, study participants raised concerns about poor enforcement of safety rules and regulations by government agencies.(36) Whilst the participants felt that this was needed, they also felt that neither party prioritised OHS; this was felt to be due to the lack of inspection and penalty or fines for infringing the rules. This study highlights the need for a co-ordinated approach to injury prevention. Through the unified efforts of government, NGOs, communities and other professionals, social inequalities, enforcement issues and educational activities could be addressed and delivered.

For peer review only

For peer review only - http://bmjopen.bmj.com/site/about/guidelines.xhtml

Strengths and limitations

To the best of our knowledge, this is the first qualitative study that captured information about home and workplace injuries among people living in varied settings and involved in various occupations in Nepal, Makwanpur district. This ensured a diversity of perspectives about both home and workplace injuries from people living in different home environments, those working in different occupations, from different levels of authority and from different socio-economic backgrounds. A limitation of the study is that it does not provide information on injury specific interventions but it does present a sound basis from which injury researchers can explore specific injury risks and identify measures to remove them.

Based on our findings, local communities appear to be receptive to home-based visits and awareness campaigns on home injury prevention. Regarding prevention of workplace injuries, our study found that there was a need for regular workplace training for both employees and employers on safety measures, along with a mechanism to ensure learning was maintained. Our study also stressed the need for a cultural shift so that greater self-efficacy to keep oneself and one's family and work colleagues safe become the norm. Findings from our study, collated with those of epidemiological studies, will be a good starting point to inform policy level discussions. This would enable formulation and enforcement of policies and strategies related to home and workplace injury prevention.

CONCLUSION

The findings of this study highlighted that both home and workplace injuries are complex and multifactorial and are influenced by personal, situational and environmental factors. Most importantly, lack of knowledge about injury risks and preventive measures, at both a community level and in the workplace were found to be common barriers to injury prevention. The introduction and implementation of educational home safety programmes delivered within the community and as an occupational safety programme within the workplace are essential. Together with previously published epidemiological evidence, the intervention programmes and the barriers and facilitators identified in this study provide a useful basis on which policy makers can base their decision-making when addressing home and workplace injuries in Nepal.

For peer review only - http://bmjopen.bmj.com/site/about/guidelines.xhtml

Footnotes

Author affiliations

1. Nepal Injury Research Centre, Kathmandu Medical College Public Limited, Kathmandu, Nepal
2. Faculty of Health and Applied Sciences, University of the West of England, Bristol, UK
3. Mother and Infant Research Activities, Kathmandu, Nepal
4. Department of Community Medicine, Kathmandu Medical College Public Limited, Kathmandu, Nepal

Acknowledgement:

We acknowledge the support of all three Municipality authorities for approving to conduct this study. We are grateful to all the staffs at Mother and Infant Research Activities who lent their 20 years of research field experience in our study supporting with recruitment of the study participants and logistics to conduct data collection. We are thankful to all the participants of our study who consented to participate and share their experience. We would like to acknowledge the support of the wider research team at NIHR Global Health Research Group at Nepal Injury Research UK and Nepal team and administrative support of Kathmandu Medical college who have supported the study.

Contributors:

SB, TD, JM, SKJ contributed to conception and designing the study. SB drafted the protocol design, methods and data analysis plan. SB and EJ contributed to data collection with support from SRM, DA. SB and EJ led analysis, interpretation of data and drafted the article. EJ and SB drafted and finalised the manuscript with equal contribution. All the authors contributed to drafts and approved the final manuscript.

Funding:

This research was funded by the National Institute for Health Research (NIHR) (Ref:16/137/49) using UK aid from the UK Government to support global health research. The views expressed are those of the author(s) and not necessarily those of the NIHR or the UK Department of Health and Social Care.

Competing interest: None declared

Patient Consent for Publication: Not required

Data Sharing Statement: Data sets are de-identified transcripts of nine interviews and nine focus groups (in Nepali translated in English). Data are available on request from corresponding author.

For peer review only

Ethics approval: Ethical approval was obtained from the Nepal Health Research Council (Reg. no. 779/2018) and the University of the West of England, Bristol, Faculty Research Ethics Committee (Reference: HAS.19.04.157). Institutional approval from each municipality office was also obtained for conducting the study in that location.

REFERENCES

1. World Health Organization. Injuries and violence: the facts 2014. Geneva, Switzerland; 2014.
2. Pant PR, Banstola A, Bhatta S*, et al.* Burden of injuries in Nepal, 1990–2017: findings from the Global Burden of Disease Study 2017. *Inj Prev*. 2020;0:1-10.DOI: http://dx.doi.org/10.1136/injuryprev-2019-043309.
3. Mock CN, Smith KR, Kobusingye O*, et al.* Injury prevention and environmental health: key messages from Disease Control Priorities. 2017.
4. Safety and health at work [Internet]. Internatioanl Labour Organization(ILO). 2020 [Accessed July 7 2020]. Available from: https://www.ilo.org/global/topics/safety-and-health-at-work/home/lang--en/index.htm.
5. Joshi SK, Shrestha S, Vaidya S. Occupational safety and health studies in Nepal. *Int J Occup Saf Health*. 2011;1(1):19-26.DOI: https://doi.org/10.3126/ijosh.v1i1.4725.
6. Butchart A, Kruger J, Lekoba R. Perceptions of injury causes and solutions in a Johannesburg township: implications for prevention. *Soc Sci Med*. 2000;50(3):331-44.DOI: https://doi.org/10.1016/S0277-9536(99)00272-5.
7. Ablewhite J, Peel I, McDaid L*, et al.* Parental perceptions of barriers and facilitators to preventing child unintentional injuries within the home: a qualitative study. *BMC Public Health*. 2015;15(1):280.DOI: https://doi.org/10.1186/s12889-015-1547-2.
8. Pant PR, Towner E, Pilkington P*, et al.* Community perceptions of unintentional child injuries in Makwanpur district of Nepal: a qualitative study. *BMC public health*. 2014;14(1):476.DOI: https://doi.org/10.1186/1471-2458-14-476.
9. Bhatta S. Community-based home injury risk assessment in rural Nepal(Thesis). UK: University of the West of England; 2017.
10. Rahman A, Shafinaz S, Linnan M*, et al.* Community perception of childhood drowning and its prevention measures in rural Bangladesh: A qualitative study. *Aust J Rural Health*. 2008;16(3):176-80.DOI: https://doi.org/10.1111/j.1440-1584.2008.00969.x.
11. Pradhan A, Poudel P, Thomas D*, et al.* A review of the evidence: suicide among women in Nepal. Kathmandu: National Health Sector Support Program, Ministry of Health and Population; 2011.
12. Akram O. Occupational health, safety and extreme poverty: a qualitative perspective from Bangladesh. *Int J Occup Saf Health*. 2014;4(1):41-50.DOI: https://doi.org/10.3126/ijosh.v4i1.10654.
13. Sapkota S, Lee A, Karki J*, et al.* Risks and risk mitigation in waste-work: a qualitative study of informal waste workers in Nepal. *Public Health in Practice*. 2020;1:100028.DOI: https://doi.org/10.1016/j.puhip.2020.100028.
14. Adhikary P, Keen S, Van Teijlingen E. Workplace accidents among Nepali male workers in the Middle East and Malaysia: A qualitative study. *J Immigr Minor Health*. 2019;21(5):1115-22.DOI: https://doi.org/10.1007/s10903-018-0801-y.
15. Gibson SK. Social learning (cognitive) theory and implications for human resource development. *Advances in developing human resources*. 2004;6(2):193-210.DOI: https://doi.org/10.1177%2F1523422304263429.
16. Braun V, Clarke V. *Successful qualitative research: A practical guide for beginners*: sage; 2013.

For peer review only

17. Maher C, Hadfield M, Hutchings M, *et al.* Ensuring rigor in qualitative data analysis: A design research approach to coding combining NVivo with traditional material methods. *Int J Qual Methods*. 2018;17(1):1609406918786362.DOI: https://doi.org/10.1177%2F1609406918786362.
18. Alonge O, Agrawal P, Talab A, *et al.* Fatal and non-fatal injury outcomes: results from a purposively sampled census of seven rural subdistricts in Bangladesh. *Lancet Glob Health*. 2017;5(8):e818-e27.DOI: https://doi.org/10.1016/S2214-109X(17)30244-9.
19. Gupta S, Gupta SK, Devkota S, *et al.* Fall injuries in Nepal: a countrywide population-based survey. *Ann Glob Health*. 2015;81(4):487-94.DOI: https://doi.org/10.1016/j.aogh.2015.07.004.
20. Bhatta S, Mytton JA, Deave T. Assessment of home hazards for non-fatal childhood injuries in rural Nepal: a community survey. *Inj Prev*. 2020.DOI: http://dx.doi.org/10.1136/injuryprev-2019-043482.
21. Khandarmaa T-O, Harun-Or-Rashid M, Sakamoto J. Risk factors of burns among children in Mongolia. *Burns*. 2012;38(5):751-7.DOI: https://doi.org/10.1016/j.burns.2011.11.006.
22. Rybarczyk MM, Schafer JM, Elm CM, *et al.* A systematic review of burn injuries in low-and middle-income countries: epidemiology in the WHO-defined African Region. *Afr J Emerg Med*. 2017;7(1):30-7.DOI: https://doi.org/10.1016/j.afjem.2017.01.006.
23. He S, Alonge O, Agrawal P, *et al.* Epidemiology of burns in rural Bangladesh: An update. *Int J Environ Res Public Health*. 2017;14(4):381.DOI: https://doi.org/10.3390/ijerph14040381.
24. Beard MR, Watson MC, Clifford MJ. Parents' perceptions of unintentional paediatric burn injuries—A qualitative study. *Burns*. 2020.DOI: https://doi.org/10.1016/j.burns.2019.12.009.
25. Munro SA, Van Niekerk A, Seedat M. Childhood unintentional injuries: the perceived impact of the environment, lack of supervision and child characteristics. *Child Care Health Dev*. 2006;32(3):269-79.DOI: https://doi.org/10.1111/j.1365-2214.2006.00593.x.
26. Hagaman AK, Khadka S, Wutich A, *et al.* Suicide in Nepal: Qualitative findings from a modified case-series psychological autopsy investigation of suicide deaths. *Cult Med Psychiatry*. 2018;42(3):704-34.DOI: https://doi.org/10.1007/s11013-018-9585-8.
27. Jagnoor J, Keay L, Jaswal N, *et al.* A qualitative study on the perceptions of preventing falls as a health priority among older people in Northern India. *Inj Prev*. 2014;20(1):29-34.DOI: http://dx.doi.org/10.1136/injuryprev-2012-040707.
28. Bakhtiyari M, Delpisheh A, Riahi SM, *et al.* Epidemiology of occupational accidents among Iranian insured workers. *Saf Sci*. 2012;50(7):1480-4.DOI: https://doi.org/10.1016/j.ssci.2012.01.015.
29. Davey S, Maheshwari C, Raghav SK, *et al.* Impact of occupational health hazards prevention messages on perceptions among rural clients in India: The outcomes of a panel study. *International Journal of Health System and Disaster Management*. 2017;5(1):11.DOI: 10.4103/ijhsdm.ijhsdm_2_17.
30. Kabir A, Farhana N, Akter F, *et al.* Sweeping practices, perceptions and knowledge about occupational safety and health hazards of street sweepers in Dhaka city, Bangladesh: a qualitative inquiry. *Int J Community Med Public Health*. 2015;2(3):237-43.DOI: http://dx.doi.org/10.18203/2394-6040.ijcmph20150476.
31. Budhathoki SS, Singh SB, Sagtani RA, *et al.* Awareness of occupational hazards and use of safety measures among welders: a cross-sectional study from eastern Nepal. *BMJ open*. 2014;4(6):e004646.DOI: http://dx.doi.org/10.1136/bmjopen-2013-004646.
32. Prasad RV, Kanimozhy K, Konduru RK, *et al.* Occupational diseases and safety measures: Perceptions of saw mill workers–A qualitative study in Pondicherry, South India. *Indian Journal of Medical Specialities*. 2016;7(1):19-22.DOI: https://doi.org/10.1016/j.injms.2015.11.002.
33. Regmi PR, Van Teijlingen E, Mahato P, *et al.* The health of Nepali migrants in India: A qualitative study of lifestyles and risks. *Int J Environ Res Public Health*. 2019;16(19):3655.DOI: https://doi.org/10.3390/ijerph16193655.
34. Carter WS. Introducing occupational health in an emerging economy: a Nepal experience. *Ann Occup Hyg*. 2010;54(5):477-85.DOI: https://doi.org/10.1093/annhyg/meq036.

For peer review only - http://bmjopen.bmj.com/site/about/guidelines.xhtml

35. Joshi SK. Occupational Safety and Health in Nepal Revisited. *Int J Occup Saf Health* 2018;8(1):1-2.DOI: https://doi.org/10.3126/ijosh.v8i1.22921.

36. Zahoor H, Chan AP, Gao R*, et al.* The factors contributing to construction accidents in Pakistan. *Engineering, construction and architectural management*. 2017;24(3):463-85.DOI: https://doi.org/10.1108/ECAM-01-2016-0027.

For peer review only

Barriers and enablers for injury prevention at home and workplace in Nepal: A qualitative study

Consolidated criteria for reporting qualitative studies (COREQ): 32-item checklist (Tong, Sainsbury & Craig, 2007)

No. Item	Guide questions	Description/Page
**Domain 1: Research team and reflexivity**		
Personal Characteristics		
1. Interviewer/facilitator	Which author/s conducted the interview or focus group?	EJ facilitated 7 Focus groups and 8 interviews and SB facilitated 2 focus groups and 1 interview Page: 4, DA helped to organize focus groups and interviews
2. Credentials	What were the researcher's credentials? E.g. PhD, MD	EJ: Masters in Nursing<br>SB: PhD<br>TD: PhD<br>JM: PhD<br>SRM: MD<br>DA: Masters in Social Science<br>SKJ: PhD
3. Occupation	What was their occupation at the time of the study?	TD, EJ, SB, JM: Researchers<br>DA: Site Coordinator<br>SKJ: Researcher, Academician<br>SRM: Researcher, Pediatrician
4. Gender	Was the researcher male or female?	TD, EJ. JM: Female<br>SB, SRM, SKJ, DA: Male
5. Experience and training	What experience or training did the researcher have?	All authors have relevant research experience and training on study design, data collection and analysis
Relationship with participants		
6. Relationship established	Was a relationship established prior to study commencement?	No relationship was established prior to study.
7. Participant knowledge of the interviewer	What did the participants know about the researcher? e.g. personal goals, reasons for doing the research	The participants were briefed about research objectives using study information sheet. Page:4
8. Interviewer characteristics	What characteristics were reported about the inter viewer/facilitator? e.g. Bias, assumptions, reasons and interests in the research topic	Trained researchers with experience in conducting injury research undertook the data collection.
**Domain 2: study design**		
Theoretical framework		
9. Methodological orientation and Theory	What methodological orientation was stated to underpin the study? e.g. grounded theory, discourse analysis, ethnography, phenomenology, content analysis	Inductive thematic analysis was done and the rationale for using it has been justified in the method section. Page:4
Participant selection		
10. Sampling	How were participants selected? e.g. purposive, convenience, consecutive, snowball	Purposive sampling. Page:3-4

For peer review only

Barriers and enablers for injury prevention at home and workplace in Nepal: A qualitative study

Consolidated criteria for reporting qualitative studies (COREQ): 32-item checklist (Tong, Sainsbury & Craig, 2007)

11. Method of approach	How were participants approached? e.g. face-to-face, telephone, mail, email	Face to face interviews and focus groups were conducted. Page:3-4
12. Sample size	How many participants were in the study?	A total of 83 in nine focus groups and nine interviews in eighteen different sessions. Page:3-4
13. Non-participation	How many people refused to participate or dropped out? Reasons?	One. No reason was given.
Setting		
14. Setting of data collection	Where was the data collected? e.g. home, clinic, workplace	The data were collected at home and workplace, whichever was convenient and feasible for the participants.
15. Presence of non-participants	Was anyone else present besides the participants and researchers?	A local field staff took notes during the focus group discussions. The field staff were not familiar with the participants.
16. Description of sample	What are the important characteristics of the sample? e.g. demographic data, date	Sex, age, participants role, location Page:4-5
Data collection		
17. Interview guide	Were questions, prompts, guides provided by the authors? Was it pilot tested?	Topic guide and interview schedule were developed for focus groups and interviews, respectively. The topic guide and interview schedule were pre-tested Page:4
18. Repeat interviews	Were repeat inter views carried out? If yes, how many?	No.
19. Audio/visual recording	Did the research use audio or visual recording to collect the data?	Audio recording of focus groups and interviews with participants consent Page:4
20. Field notes	Were field notes made during and/or after the interview or focus group?	Brief field and observation notes were made during focus groups.
21. Duration	What was the duration of the inter views or focus group?	The average focus groups were 37 minutes and interviews were 31 minutes.
22. Data saturation	Was data saturation discussed?	No. Not required as per the study aim
23. Transcripts returned	Were transcripts returned to participants for comment and/or correction?	The transcripts were not returned to participants.
**Domain 3: analysis and findings**		
Data analysis		
24. Number of data coders	How many data coders coded the data?	Two
25. Description of the coding tree	Did authors provide a description of the coding tree?	No. Can be made available if requested
26. Derivation of themes	Were themes identified in advance or	Identified from the data.

For peer review only

Barriers and enablers for injury prevention at home and workplace in Nepal: A qualitative study

Consolidated criteria for reporting qualitative studies (COREQ): 32-item checklist (Tong, Sainsbury & Craig, 2007)

	derived from the data?	
27. Software	What software, if applicable, was used to manage the data?	Translation, transcription, and data analysis were done manually with the use of NVivo to manage the data.
28. Participant checking	Did participants provide feedback on the findings?	The participants didn't provide feedback on the findings.
Reporting		
29. Quotations presented	Were participant quotations presented to illustrate the themes/findings? Was each quotation identified? e.g. participant number	The quotes from participants were used and identified using a personal identification code to ensure anonymity. Page:6-8
30. Data and findings consistent	Was there consistency between the data presented and the findings?	There was consistency between the data presented and the findings.
31. Clarity of major themes	Were major themes clearly presented in the findings?	Yes. Major themes are described. Page: 4-9
32. Clarity of minor themes	Is there a description of diverse cases or discussion of minor themes?	We presented the major themes. Diversity of the participants are described. Page: 4 & 5

For peer review only

BMJ Open

Perceptions of injury risk in the home and workplace in Nepal: a qualitative study

Journal:	*BMJ Open*
Manuscript ID	bmjopen-2020-044273.R1
Article Type:	Original research
Date Submitted by the Author:	29-Jan-2021
Complete List of Authors:	Joshi, Elisha; Kathmandu Medical College and Teaching Hospital, Community Medicine<br>Bhatta, Santosh; University of the West of England, Faculty of Health and Applied Sciences<br>Deave, Toity; University of the West of England, Bristol, Faculty of Health and Applied Sciences<br>Mytton, Julie; University West of England, Faculty of Health and Applied Sciences<br>Adhikari, Dhruba; Mother and Infant Research Activities<br>Manandhar, Sunil Raja; Mother and Infant Research Activities<br>Joshi, Sunil Kumar; Kathmandu Medical College and Teaching Hospital, Community Medicine
**Primary Subject Heading**:	Qualitative research
Secondary Subject Heading:	Public health, Sociology, Health policy, Evidence based practice
Keywords:	QUALITATIVE RESEARCH, PUBLIC HEALTH, Health & safety < HEALTH SERVICES ADMINISTRATION & MANAGEMENT, PREVENTIVE MEDICINE, SOCIAL MEDICINE

SCHOLARONE™
Manuscripts

I, the Submitting Author has the right to grant and does grant on behalf of all authors of the Work (as defined in the below author licence), an exclusive licence and/or a non-exclusive licence for contributions from authors who are: i) UK Crown employees; ii) where BMJ has agreed a CC-BY licence shall apply, and/or iii) in accordance with the terms applicable for US Federal Government officers or employees acting as part of their official duties; on a worldwide, perpetual, irrevocable, royalty-free basis to BMJ Publishing Group Ltd ("BMJ") its licensees and where the relevant Journal is co-owned by BMJ to the co-owners of the Journal, to publish the Work in this journal and any other BMJ products and to exploit all rights, as set out in our licence.

The Submitting Author accepts and understands that any supply made under these terms is made by BMJ to the Submitting Author unless you are acting as an employee on behalf of your employer or a postgraduate student of an affiliated institution which is paying any applicable article publishing charge ("APC") for Open Access articles. Where the Submitting Author wishes to make the Work available on an Open Access basis (and intends to pay the relevant APC), the terms of reuse of such Open Access shall be governed by a Creative Commons licence – details of these licences and which Creative Commons licence will apply to this Work are set out in our licence referred to above.

Other than as permitted in any relevant BMJ Author's Self Archiving Policies, I confirm this Work has not been accepted for publication elsewhere, is not being considered for publication elsewhere and does not duplicate material already published. I confirm all authors consent to publication of this Work and authorise the granting of this licence.

For peer review only

Perceptions of injury risk in the home and workplace in Nepal: a qualitative study

Corresponding Author

1. Elisha Joshi*

Nepal Injury Research Centre, Kathmandu Medical College, Duwakot, Bhaktapur, Nepal
ejoshi03@gmail.com; https://orcid.org/0000-0002-3534-276X

Co-Authors

2. Santosh Bhatta*

Faculty of Health and Applied Sciences, University of the West of England, Bristol, UK
https://orcid.org/0000-0001-6393-2495

**Elisha Joshi and Santosh Bhatta have contributed equally*

3. Toity Deave

Faculty of Health and Applied Sciences, University of the West of England, Bristol, UK
https://orcid.org/0000-0001-9966-8948

4. Julie Mytton

Faculty of Health and Applied Sciences, University of the West of England, Bristol, UK
http://orcid.org/0000-0002-0306-4750

5. Dhruba Adhikari

Mother and Infant Research Activities (MIRA), Makwanpur, Nepal

6. Sunil Raja Manandhar

Kathmandu Medical College and Mother and Infant Research Activities (MIRA), Makwanpur, Nepal
https://orcid.org/0000-0002-6583-3973

7. Sunil Kumar Joshi

Department of Community Medicine, Kathmandu Medical College Public Limited, Kathmandu, Nepal
https://orcid.org/0000-0002-2704-5060

Word Count: 3966 (Max 4000 words)

For peer review only - http://bmjopen.bmj.com/site/about/guidelines.xhtml

ABSTRACT

Objective: Injuries are a global health problem. To develop context specific injury prevention interventions, one needs to understand population perceptions of home and workplace injuries. This study explored a range of views about the perception of injuries in a variety of settings and identified barriers and facilitators to injury prevention.

Design: Qualitative study: interviews and focus groups.

Setting: Three municipalities: Hetauda sub-metropolitan city, Thaha municipality (urban) and Bakaiya rural municipality in the Makwanpur, Nepal.

Participants: Nine focus groups (74 participants) and nine one-to-one interviews were completed; workers from diverse occupations, residents (slum, traditional or modern homes), health workers and local government decision makers participated in the study between May and August 2019. The interviews and discussions were audio-recorded, transcribed verbatim, translated into English and analysed thematically.

Results: Six themes were developed: unsafe home and workplace environment; inadequate supervision and monitoring; injuries are inevitable; safety takes low priority- financial and behavioural considerations; safety education and training; government-led safety programmes and enforcement. Key barrier to injury prevention was perceived to be lack of knowledge about injury risk and preventive measures at both a community level and in the workplace. Facilitators were community level educational programmes and health and safety training to employees and employers. Participants stressed the importance of the role of government in planning future injury prevention programmes in different environments.

Conclusions: This study highlighted that both home and workplace injuries are complex and multifactorial. Most importantly, lack of knowledge about injury risks and preventive measures, at both community level and in the workplace, were found to be common barriers to injury prevention; perceived to be mitigated by educational programmes. Together with previously published epidemiological evidence, the barriers and facilitators identified in this study offers useful basis to inform policy and practice.

Keywords: Perception, injury risk, home, workplace, qualitative, Nepal

Strengths and limitations of this study

- Participants from diverse home environments, different work settings and different socio-economic backgrounds yielded a breadth of views.
- This is the first study to have explored qualitatively the views of the public on perceptions of injury risks at home and at work in Nepal.
- The study is not able to provide perceptions about injury risks and preventive measures by injury type.

For peer review only - http://bmjopen.bmj.com/site/about/guidelines.xhtml

INTRODUCTION

Injuries are a global health problem, although being predictable and largely preventable.(1) According to the global estimate, nearly 4.5 million people died from injuries in 2017, with a rate of disability-adjusted life years (DALYs) of 3267 per 100,000.(2) Ninety-percent of injury-related deaths occur in low- and middle-income countries (LMICs) and, in Nepal, there has been an increase in injury-related deaths from 6.3% to 9.2% between 1990 and 2017.(3) Globally, road traffic injuries, falls, burns, poisonings and suicides are the leading causes for unintentional and intentional injuries.(4) According to the International Labour Organization, more than 2.78 million deaths per year are estimated to be due to occupational injuries or workplace disease.(5) In Nepal, 200 workers die and 20,000 workers suffer from workplace injuries yearly.(6)

Recent evidence for injuries occurring at home,(7) identified parental supervision and teaching children about injury risks were as facilitators, while barriers to child injury prevention were identified as parents' lack of anticipation of injury risks and perceiving injuries as inevitable events.(8) Culturally acceptable prevention measures, appropriate supervision arrangements, separation of hazards and training children and parents about safety were suggested by a study of community perceptions in Makwanpur, Nepal.(9) Two community-based studies conducted in rural Nepal emphasised that unintentional child injuries were thought to be due to coincidence, bad luck, witchcraft or ill fate.(9, 10) Likewise, in Bangladesh, child drowning was believed to be a result of ill fate and unpreventable.(11) Rarely were the environmental and infrastructural factors thought by parents to be the cause of child injury.(9)

Workplace injuries are becoming a public health concern in all LMICs. One qualitative study undertaken in Bangladesh found that poor people were at greater risk of injury, employers were reluctant to take responsibility for workers and subcontracting workers was observed to increase the injury risk.(12) Despite the high level of awareness about the use of personal protective equipment (PPE) among Nepali workers, there was poor practice of using PPE.(13) A qualitative study conducted among Nepali migrant workers suggested that workplace injuries were due to lack of health and safety regulations, the risk-taking behaviour of workers and perceived work pressure.(14)

Human behaviour, being a complex phenomenon, is determined by environmental factors (such as social support/barriers, ability to change one\s own environment), behavioural factors (such as skills, practice and self-efficacy) and personal factors (such as knowledge and, perception).(15) To understand health and safety behaviours, one needs to understand how people perceive injury risks and what the factors are that influence their behaviours.(13) Little is known about how the people of Nepal perceive and deal with home and workplace injuries or their risk factors. This study explored a

For peer review only - http://bmjopen.bmj.com/site/about/guidelines.xhtml

range of views about the perception of injuries in a variety of settings and identified barriers and facilitators to injury prevention.

METHODS

Study design

We adopted a qualitative research methodology using focus group discussions and key informant interviews.

Study setting and participants

The study took place in Makwanpur district of Nepal (See Figure 1) that has a mixed topography similar to other districts in the country. The three administrative areas (‘*palikas*’) were selected purposively: Hetauda sub-metropolitan city (urban area), Thaha municipality (suburban area) and Bakaiya municipality (rural area). To ensure diversity of location, occupation, housing type and key government personnel, and to achieve the information power necessary to answer the research question,(16) the research team prepared a pre-specified sampling framework where we identified and listed key groups we wished to include. Members of the research team consulted with the existing networks and local government officers to identify the knowledgeable and experienced individuals and groups of participants. In each study area, a local non- governmental organisation (NGO), Mother and Infant Research Activities (MIRA), who have over 20 years of experience of working in Makwanpur district, Nepal, helped to identify prospective participants. The participants who met the inclusion criteria, were 18 years old or over and resident and/or working in one of the three *palikas*, were approached by the research team. Identified individuals were invited to take part, were given a participant information sheet and consent form. Consent forms were completed for those who agreed to take part. The research team conducted all the focus groups and interviews at a location and time convenient to the participant groups and individuals in each study site. There was no adverse consequence for non-participation and as a token of appreciation for their time and participation, each participant was given 1000 Nepali Rupees (equivalent to approximately 6 British Pounds).

For peer review only - http://bmjopen.bmj.com/site/about/guidelines.xhtml

Data collection

The focus groups (FGs) and interviews were conducted to explore views and issues of people residing in varied accommodation and environments and workers in their different workplaces. Interviews were conducted with key people, based on their role and nature of their job.

A topic guide for the focus groups and a semi-structured interview schedule were developed (see supplementary files). These were developed based on previous work undertaken by the research team, pretested and finalised prior to data collection. All focus groups and interviews were conducted face-to-face by either SB or EJ. With consent, the focus groups and interviews were digitally audio-recorded and written notes were taken concurrently by a note taker to capture non-verbal communication. The interview schedule and topic guide asked questions about, for example, perception of injuries, risk factors and barriers and facilitators to injury prevention.

Data management

Recordings were transcribed verbatim in Nepali and then translated into English with an aim to retain the original meaning of the statements. The transcriptions and translations were completed by experienced Mother and Infant Research Activities (MIRA) staff and verified by researchers (EJ & SB). Personal identifiable information from participants were removed from transcriptions and replaced with a unique identification code.

Data analysis

The interviews and focus groups data were analysed using inductive thematic analysis to search for repeated patterns of meaning or themes in the data.(17) NVivo qualitative data analysis software, Version 12 was used to arrange the codes systematically and collate data relevant to each code.(18) Two transcripts were coded by both EJ and SB and discussed with a third researcher (TD) to agree an initial coding framework which was then applied consistently across the transcripts. After coding all transcripts, the individual codes were reviewed and placed into clusters based on their similarities. The clusters were organised to develop candidate themes and barriers and facilitators were identified. Any discrepancies and difference in codes and themes developed were discussed with a third researcher (TD) and final themes were agreed.

Patient and Public Involvement

We worked with a local NGO (MIRA) and local government officials to identify relevant groups and key personnel to recruit as participants in our study. No patients were involved in this study.

For peer review only - http://bmjopen.bmj.com/site/about/guidelines.xhtml

RESULTS

Participants' characteristics

We conducted nine focus groups and nine one-to-one interviews with a total of 83 participants across three municipalities. One participant refused to participate in the interview, participation was 100%. The average length of each focus group was 37 minutes and interview was 31 minutes. A total of six focus groups and six interviews were conducted in Hetauda sub-metropolitan city, two focus groups and two interviews in Bakaiya rural municipality and one focus groups and one interview in Thaha municipality. Participants in the focus groups were from similar backgrounds (table 1) whereas interviewees were representatives of different institutes and organisations (table 2).

Table 1. Characteristics of the focus group participants

Focus Group ID	Participants	Participant numbers	Age range of participants (years)	Gender
FG 1	Residents in rural, traditionally built home	8	21-55	M:4<br>F:4
FG 2	Residents in rural, concrete/modern home	9	21-55	M:5<br>F:4
FG 3	Residents in urban area	8	35-50	M:4<br>F:4
FG 4	Haulage/truck drivers	9	40-57	M:9
FG 5	Residents in slum area	8	18-59	M:4<br>F:4
FG 6	Skilled construction workers: plumber, welder, electrician	8	21-50	M:8
FG 7	Farmers (agriculture, animal husbandry)	8	37-51	M:4<br>F:4
FG 8	Factory workers (cement factory)	9	26-41	M:8<br>F:1
FG 9	Trade professionals	7	30-53	M:7

Male: M, Female: F; FG: Focus Group;

Table 2. Characteristics of the interview participants

Interviewee ID	Participants	Age range of participants (years)	Gender
KII 1	President of mothers' group	40-50	F
KII 2	Consultant in charge of hospital emergency department	20-30	F
KII 3	Healthcare Manager of municipality	40-50	M
KII 4	Healthcare Manager of sub-metropolitan city	40-50	F
KII 5	Trade union representatives	50-60	M
KII 6	Senior Officer of Hetauda industrial area	40-50	M

For peer review only - http://bmjopen.bmj.com/site/about/guidelines.xhtml

KII 7	Representative of transport workers	40-50	M
KII 8	Rural municipality officer	40-50	M
KII 9	Senior executive of education	50-60	M

KII: Key Informant Interview

Themes

Six themes were identified from the focus groups and interview data and these are described below. Relevant quotes are presented to illustrate the key themes.

1. Unsafe home and workplace environment

The physical infrastructure and home environment were perceived as common causes of unintentional injuries at home. The participants said that the lack of railings on stairs was the leading cause of fall injuries among people living in traditionally built rural houses, especially hazardous to children. Participants from urban areas raised their concerns that elderly people sustained fall injuries due to slipping on marble or tiled bathroom floors. Burn injuries were common among children and women where firewood was used for cooking.

> *While the kids are running in the balcony, they jump from there and get hurt. It's like that in these mud houses...there is no fence[railing]!" (FGD_1, P8)*

Regarding workplace injuries, different employees, like factory workers, healthcare staff and farmers all highlighted that falls, cuts and burns were common injuries. Some described how they have to work in risky environments sometimes without safety equipment. One factory worker reported:

> *I have seen workers suffering from the pain due to heat. They have to take out rods from 300 degrees of heat by bare hands. They even don't wear gloves and boots. They use scissors [instead of tongs] ….* (KII 6)

Many participants explained that workers who are illiterate and suffer from poverty were those who worked under private contractors who are less likely to provide safety equipment.

2. Inadequate supervision and monitoring

The participants highlighted the need for close supervision and monitoring of children and teenagers. One of the focus groups reported that there had been incidents of poisoning when children find liquids that look like food or drink. Although parents said they were aware of this, they said that careful supervision was difficult for working and single parents and, therefore, injuries occurred.

For peer review only - http://bmjopen.bmj.com/site/about/guidelines.xhtml

The parents have to work throughout the day to earn food and the children are guided either by the school or without anyone proper guidance. The parents are unknown about their children's behaviour. They simply grow without their parents' guidance... (KII 9)

3. Injuries are inevitable

In regards to both home and workplace injuries, participants highlighted that injuries occur by chance, unexpectedly and are normal in their situation. Some participants felt that one's luck is responsible for occurrence of injuries. Acceptance of injuries as a normal part of life was a predominant finding; they were uncertain about how injuries could be prevented. They believed that such injuries that occurred abruptly could not be prevented, despite them being careful when performing tasks.

I feel that people get injured if they have misfortune or if the movement of planets that influences the destinies of people is not good or else, they [people] won't get injured. Even if we are careful, we might get injured due to our bad fortune. (FG 5, P2)

It's like this. In the workshops the cuts are normal. We need to play with the metals. While doing that [playing with metals] the sharp edges of it [metals] can give cuts to us. We also get normal cuts when we are cutting down the pipes. (FGD_6_P2)

4. Safety takes low priority: financial and behavioural considerations

Most participants said that when they were at home they didn't think about safety. Carelessness when completing tasks in a hurry was the most common reason for injuries occurring at home and workplace.

Most of the injuries take place due to our carelessness. Like we throw nails and other sharp materials here and there…. Another reason is … performing any kind of activities hurriedly while walking … (FG 2, P2)

Participants from rural villages confessed that financial constraints hindered them from implementing safety measures like installing railings to balconies as they struggled to meet their families' basic needs.

Due to this economic barrier, we are unable to manage everything… (FG 1, P5)

Participants described the common practice of employers who pressurised their workers to work fast without providing PPE. Inadequate capital was one reason for employers for not providing PPE or installing safer, automatic machines.

For peer review only - http://bmjopen.bmj.com/site/about/guidelines.xhtml

> *…the manufacturers of construction factories like grill factories are more concerned about how to increase their production leading the workers to work fast and carelessness at work.* (KII 5)

The workers stated that, not only did they have to work without PPE, especially the contracted workers, but most of them worked carelessly, misusing or not using safety equipment even when the PPE was available. Overconfidence and feeling that nothing would happen to them led the workers to take such risks.

> *Even if we have safety equipment, we get careless and do not use them thinking that the work we are about to perform just takes 2 minutes of time and we won't require it [safety equipment]. But, in that 2 minutes of time any incident may occur.* (FG 7, P3)

Senior personnel emphasized that the work supervisors should consider safety, raise awareness and orient the workers to the dangerous aspects of their work.

> *What I would say is once you recruit the person, he/she should be taught everything. Every industry should manage this and conduct orientation. The industry should make employees learn about the culture of work. But this culture isn't practised.* (KII 6)

5. Safety Education and Training

Additional to the behavioural and financial aspects, participants reported that lack of awareness was a major contributing factor that led to home and workplace injury; illiteracy and a lack of understanding about injury was prevalent. Almost all the participants voiced the need for an awareness programme, that it would be a crucial enabling factor for injury prevention, both in the home and workplace.

> *In case of home, there should be awareness program because they [community people] don't know. They should be educated such that they can bring change in their behaviour. It is because they don't understand.* (KII 3)

Regarding home injury, many participants stated that a programme to reduce injuries at home should be conducted by local community groups and their leaders, such as the mothers' groups. Participants believed that effectiveness of programmes relied on being led by injury experts in collaboration with NGOs, International Non- Governmental Organisations (INGOs) and local government.

> *If the outsiders go and tell them [community people] they might listen. But if we locals tell them they won't listen to us.* (FG2, P4)

For peer review only - http://bmjopen.bmj.com/site/about/guidelines.xhtml

Participants also felt that such injury prevention programmes should be delivered to school children, teachers, parents and community leaders, using social media or community mobilisation.

> *For that we must gather all the family members in a place and discuss about this matter [injuries]. We must discuss about how injuries take place and how we can reduce it [injuries]....* (FG 3, P7)

Factory workers raised concerns about the lack of institute to train workers, to make them aware of occupational health and safety. Skilled workers and laborers highlighted the need for safety training, that it could be provided and monitored by industries, NGOs and the Ministry of Labour. There was a consensus among factory workers that training needed to be inclusive of all workers, company and contract staff, including the employers, that it should be done regularly, addressing any technological updates and human tendency to forget. Pamphlets or brochures with brief messages would raise awareness amongst the workers.

> *The main important thing is to [make aware] the employer, those who employ the workers. Basically, they should be aware of the health of the workers. We should also be able to make the workers understand that, if you are healthy then you can do lot of work...* (KII 5)

6. Government led safety programmes and enforcement

Almost all participants thought that the government's role was pivotal for injury prevention activities. They thought that government lacked focus on injury prevention activities due to the lack of injury data. Most participants believed that the local government authorities needed to take a lead on injury prevention activities. On probing what would work, they thought that with NGOs, INGOs and injury experts collaborating, strategic planning could be achieved. Participants provided examples of how the municipality was working in partnership with some organisations for community health development and women empowerment. For example, the municipality was working with research organisations to prioritise injury prevention programmes.

> *The local government along with the NGO/INGOs have mobilized the mother's group... The local government should launch such a program [injury prevention] which will enhance them [mothers] and help to promote the injury related activities.* (KII 9)

The participants highlighted the role of central government could be to formulate uniform policies, with strict enforcement of rules and regulations to prevent injuries both in the home and workplace. Periodic monitoring visits to industries undertaken by the government and private sectors was felt to be mandatory to ensure safety standards were being followed.

For peer review only - http://bmjopen.bmj.com/site/about/guidelines.xhtml

The government of Nepal should formulate standard protocol to prevent injuries at industries. If there isn't environment as mentioned in the protocol then they shouldn't be renewed and the industry should be closed immediately. (KII 3)

Barriers and facilitators to injury prevention

The key barrier to injury prevention identified by participants was the lack of knowledge about injury risk and preventive measures, at both a community level and in the workplace. Other barriers included: safety not being a priority, and the inability to improve the safety of the home or working environments due to financial constraints. The barriers, particularly for child injury prevention, included the inability of parents to provide adequate supervision. Participants identified that safety at home and work could be improved at a community level, through educational programmes to raise awareness about how injuries could be prevented. In the workplace, health and safety training could be provided for employers and employees and safety laws introduced with rigorous enforcement. The involvement of external agencies and involvement of government authorities could facilitate the implementation and effectiveness of such programmes, training and legislation.

DISCUSSION

This study found that injuries were perceived as a problematic issue and preventive measures have been neglected across different settings.

Home injuries

In our study, the home environment and socioeconomic aspects were highlighted as the main contributing factors for injuries such as falls, burns and poisonings. Unsafe buildings and cooking on open fires have been identified previously as injury hazards(19) and, in a countrywide population-based study conducted in Nepal, falls were found to be the leading cause of injury.(20) A household survey that assessed home hazards for child injury in rural Nepal, found that 98% of households did not have protective railings on stairs, more than 80% households had no window bars and 50% households lacked a protective barrier on their balconies.(21) Similar to the findings from Bangladesh, the study participants perceived their financial constraints to be one of the barriers to prevention of home injuries.(22)

The findings from our study supports those from previous epidemiological evidence where children were found to be at high risk of burns and that the home was hazardous.(23, 24) Participants in our study provided the context and reported reasons for some of the more common injuries identified

For peer review only - http://bmjopen.bmj.com/site/about/guidelines.xhtml

by other studies. For example, open fires for cooking was unsafe(25) and the majority of households (61%) in rural areas of Nepal had chemicals or fertilisers within the reach of children.(21)

Some parents believed that injuries were a normal part of child development, bad luck, witchcraft or ill fate. This fatalism, in relation to injury, has been reported in previous studies conducted in LMICs(7, 26, 27) but no method to address this belief has been developed. A qualitative study in Nepal showed that when people are unable to rationalize the cause then the concept of fate is ascribed.(28) This lack of knowledge in the community has been reported in previous studies conducted in Nepal.(9, 10) In line with previous studies, the participants of this study also highlighted the need for interventions at all levels, such as government, NGOs and the local community.(22)

Workplace injuries

Minor injuries were reported as being common occurrences within the workplace but fatal injuries and those that led to long-term disability were also reported. Study participants believed that poverty and illiteracy were interlinked and were the root cause of workplace injuries. This might be because employers tended to hire the poorer workers who are ready to take on risky jobs but with a relatively higher wage(12) or that, similar to our study, carelessness was a significant causal factor, as found in one study of Iranian workers.(29)

Study participants stressed the need to raise awareness amongst workers about the importance of PPE use via regular and periodic occupational health and safety education programmes. A study in India demonstrated that the understanding of occupational hazards was significantly associated with the literacy status of workers and delivering occupational health education programmes augmented the understanding of Occupational Health and Safety (OHS).(30) Another study suggested that such programmes should be delivered together with behaviour change counselling(31) but our study participants believed that awareness programmes, with adequate PPE and enforcement of occupational standards, would suffice for adopting safe workplace practices. One study found that high levels of awareness of occupational hazards and use of PPE did not lead to the actual use of PPE.(32) Further exploration is needed about why workers don’t use PPE despite the availability and training. Unlike other studies, which highlighted lack of user-friendly PPE and ineffective PPE(33, 34), our study found both an absence of formal training prior to commencing work and lack of PPE which created environments conducive to injuries.

For peer review only

For peer review only - http://bmjopen.bmj.com/site/about/guidelines.xhtml

In Nepal, the 1992 Occupational Health and Safety law did not specify how to evaluate or enforce the law.(35) After the revision in 2017, it explicitly described the roles of employees and employers in adhering to OHS standards, yet legislation is silent on the supervision and monitoring of the implementation status.(36) In line with other studies, study participants raised concerns about poor enforcement of safety rules and regulations by government agencies.(37) Whilst the participants felt that this was needed, they also felt that neither party prioritised OHS; this was felt to be due to the lack of inspection and lack of enforcing penalty or fines for infringing the rules. This study highlights the need for a co-ordinated approach to injury prevention. Through the unified efforts of government, NGOs, communities and other professionals, social inequalities, enforcement issues and educational activities could be addressed and delivered.

Strengths and limitations

To the best of our knowledge, this is the first qualitative study that has reported information about home and workplace injuries among people living in varied settings and involved in various occupations in Nepal, Makwanpur district. The study design ensured a diversity of perspectives about both home and workplace injuries from people living in different home environments, those working in different occupations, from different levels of authority and from different socio-economic backgrounds. A limitation of the study is that it does not provide information on injury specific interventions but it does present a sound basis from which injury researchers can explore specific injury risks and identify measures to remove them.

Future Directions and Possible solutions

Based on participant's suggestions, home-based visits and awareness campaigns could be potential interventions for home injury prevention. Regarding prevention of workplace injuries, our study found that there was a need for regular workplace training for both employees and employers on safety measures, along with a mechanism to ensure levels of knowledge were maintained. Our study also stressed the need for a cultural shift so that greater self-efficacy to keep oneself and one's family and work colleagues safe becomes the norm. Findings from our study, collated with those of epidemiological studies, will be a good starting point to inform policy-level discussions. This would enable formulation and enforcement of policies and strategies related to home and workplace injury prevention.

CONCLUSION

The findings of this study highlighted that both home and workplace injuries are complex and multifactorial and are influenced by personal, situational and environmental factors. Most

For peer review only - http://bmjopen.bmj.com/site/about/guidelines.xhtml

importantly, lack of knowledge about injury risks and preventive measures, at both a community level and in the workplace were found to be common barriers to injury prevention. The introduction and implementation of educational home safety programmes delivered within the community and as an occupational safety programme within the workplace would be welcomed. Together with previously published epidemiological evidence, the perceptions of risk and perceived barriers and facilitators to safety identified in this study provide a useful basis on which policy makers can establish their decision-making when addressing home and workplace injuries in Nepal.

Footnotes

Author affiliations

1. Nepal Injury Research Centre, Kathmandu Medical College Public Limited, Kathmandu, Nepal
2. Faculty of Health and Applied Sciences, University of the West of England, Bristol, UK
3. Mother and Infant Research Activities, Kathmandu, Nepal
4. Department of Community Medicine, Kathmandu Medical College Public Limited, Kathmandu, Nepal

Acknowledgement:

We acknowledge the support of all three Palika authorities for approving to conduct this study. We are grateful to all the staffs at Mother and Infant Research Activities who lent their 20 years of research field experience to our study supporting recruitment of the study participants and logistics to conduct data collection. We are thankful to all the participants of our study who consented to participate and share their experience. We would like to acknowledge the support of the wider research team at NIHR Global Health Research Group at Nepal Injury Research UK (especially Emer Brangan) and Nepal team and administrative support of Kathmandu Medical college who have supported the study.

For peer review only

Contributors:

SB, TD, JM, SKJ contributed to conception and designing the study. SB drafted the protocol design, methods and data analysis plan. SB and EJ contributed to data collection with support from SRM, DA. SB and EJ led analysis, interpretation of data and drafted the article. EJ and SB drafted and finalised the manuscript with equal contribution. All the authors contributed to drafts and approved the final manuscript.

Funding:

For peer review only - http://bmjopen.bmj.com/site/about/guidelines.xhtml

This research was funded by the National Institute for Health Research (NIHR) (Ref:16/137/49) using UK aid from the UK Government to support global health research. The views expressed are those of the author(s) and not necessarily those of the NIHR or the UK Department of Health and Social Care.

Competing interest: None declared

Patient Consent for Publication: Not required

Data Sharing Statement: Data sets are de-identified transcripts of nine interviews and nine focus groups (in Nepali translated in English). Data are available on request from corresponding author.

Ethics approval: Ethical approval was obtained from the Nepal Health Research Council (Reg. no. 779/2018) and the University of the West of England, Bristol, Faculty Research Ethics Committee (Reference: HAS.19.04.157). Institutional approval from each municipality office was also obtained for conducting the study in that location.

REFERENCES

1. World Health Organization. Injuries and violence: the facts 2014. Geneva, Switzerland; 2014.
2. James SL, Castle CD, Dingels ZV*, et al.* Global injury morbidity and mortality from 1990 to 2017: results from the Global Burden of Disease Study 2017. *Inj Prev*. 2020.DOI: http://dx.doi.org/10.1136/injuryprev-2019-043494.
3. Pant PR, Banstola A, Bhatta S*, et al.* Burden of injuries in Nepal, 1990–2017: findings from the Global Burden of Disease Study 2017. *Inj Prev*. 2020;0:1-10.DOI: http://dx.doi.org/10.1136/injuryprev-2019-043309.
4. Mock CN, Smith KR, Kobusingye O*, et al.* Injury prevention and environmental health: key messages from Disease Control Priorities. 2017.
5. Safety and health at work [Internet]. Internatioanl Labour Organization(ILO). 2020 [Accessed July 7 2020]. Available from: https://www.ilo.org/global/topics/safety-and-health-at-work/home/lang--en/index.htm.
6. Joshi SK, Shrestha S, Vaidya S. Occupational safety and health studies in Nepal. *Int J Occup Saf Health*. 2011;1(1):19-26.DOI: https://doi.org/10.3126/ijosh.v1i1.4725.
7. Butchart A, Kruger J, Lekoba R. Perceptions of injury causes and solutions in a Johannesburg township: implications for prevention. *Soc Sci Med*. 2000;50(3):331-44.DOI: https://doi.org/10.1016/S0277-9536(99)00272-5.
8. Ablewhite J, Peel I, McDaid L*, et al.* Parental perceptions of barriers and facilitators to preventing child unintentional injuries within the home: a qualitative study. *BMC Public Health*. 2015;15(1):280.
9. Pant PR, Towner E, Pilkington P*, et al.* Community perceptions of unintentional child injuries in Makwanpur district of Nepal: a qualitative study. *BMC public health*. 2014;14(1):476.
10. Bhatta S. Community-based home injury risk assessment in rural Nepal(Thesis). UK: University of the West of England; 2017.
11. Rahman A, Shafinaz S, Linnan M*, et al.* Community perception of childhood drowning and its prevention measures in rural Bangladesh: A qualitative study. *Aust J Rural Health*. 2008;16(3):176-80.DOI: https://doi.org/10.1111/j.1440-1584.2008.00969.x.
12. Akram O. Occupational health, safety and extreme poverty: a qualitative perspective from Bangladesh. *Int J Occup Saf Health*. 2014;4(1):41-50.DOI: https://doi.org/10.3126/ijosh.v4i1.10654.

For peer review only - http://bmjopen.bmj.com/site/about/guidelines.xhtml

13. Sapkota S, Lee A, Karki J, *et al.* Risks and risk mitigation in waste-work: a qualitative study of informal waste workers in Nepal. *Public Health in Practice*. 2020;1:100028.DOI: https://doi.org/10.1016/j.puhip.2020.100028.
14. Adhikary P, Keen S, Van Teijlingen E. Workplace accidents among Nepali male workers in the Middle East and Malaysia: A qualitative study. *J Immigr Minor Health*. 2019;21(5):1115-22.DOI: https://doi.org/10.1007/s10903-018-0801-y.
15. Gibson SK. Social learning (cognitive) theory and implications for human resource development. *Advances in developing human resources*. 2004;6(2):193-210.
16. Malterud K, Siersma VD, Guassora AD. Sample size in qualitative interview studies: guided by information power. *Qual Health Res*. 2016;26(13):1753-60.DOI: https://doi.org/10.1177%2F1049732315617444.
17. Braun V, Clarke V. *Successful qualitative research: A practical guide for beginners*: sage; 2013.
18. Maher C, Hadfield M, Hutchings M, *et al.* Ensuring rigor in qualitative data analysis: A design research approach to coding combining NVivo with traditional material methods. *Int J Qual Methods*. 2018;17(1):1609406918786362.DOI: https://doi.org/10.1177%2F1609406918786362.
19. Alonge O, Agrawal P, Talab A, *et al.* Fatal and non-fatal injury outcomes: results from a purposively sampled census of seven rural subdistricts in Bangladesh. *Lancet Glob Health*. 2017;5(8):e818-e27.DOI: https://doi.org/10.1016/S2214-109X(17)30244-9.
20. Gupta S, Gupta SK, Devkota S, *et al.* Fall injuries in Nepal: a countrywide population-based survey. *Ann Glob Health*. 2015;81(4):487-94.DOI: https://doi.org/10.1016/j.aogh.2015.07.004.
21. Bhatta S, Mytton JA, Deave T. Assessment of home hazards for non-fatal childhood injuries in rural Nepal: a community survey. *Inj Prev*. 2020.DOI: http://dx.doi.org/10.1136/injuryprev-2019-043482.
22. Jagnoor J, Keay L, Jaswal N, *et al.* A qualitative study on the perceptions of preventing falls as a health priority among older people in Northern India. *Inj Prev*. 2014;20(1):29-34.DOI: http://dx.doi.org/10.1136/injuryprev-2012-040707.
23. Khandarmaa T-O, Harun-Or-Rashid M, Sakamoto J. Risk factors of burns among children in Mongolia. *Burns*. 2012;38(5):751-7.DOI: https://doi.org/10.1016/j.burns.2011.11.006.
24. Rybarczyk MM, Schafer JM, Elm CM, *et al.* A systematic review of burn injuries in low-and middle-income countries: epidemiology in the WHO-defined African Region. *Afr J Emerg Med*. 2017;7(1):30-7.DOI: https://doi.org/10.1016/j.afjem.2017.01.006.
25. He S, Alonge O, Agrawal P, *et al.* Epidemiology of burns in rural Bangladesh: An update. *Int J Environ Res Public Health*. 2017;14(4):381.DOI: https://doi.org/10.3390/ijerph14040381.
26. Beard MR, Watson MC, Clifford MJ. Parents' perceptions of unintentional paediatric burn injuries—A qualitative study. *Burns*. 2020.DOI: https://doi.org/10.1016/j.burns.2019.12.009.
27. Munro SA, Van Niekerk A, Seedat M. Childhood unintentional injuries: the perceived impact of the environment, lack of supervision and child characteristics. *Child Care Health Dev*. 2006;32(3):269-79.DOI: https://doi.org/10.1111/j.1365-2214.2006.00593.x.
28. Hagaman AK, Khadka S, Wutich A, *et al.* Suicide in Nepal: Qualitative findings from a modified case-series psychological autopsy investigation of suicide deaths. *Cult Med Psychiatry*. 2018;42(3):704-34.DOI: https://doi.org/10.1007/s11013-018-9585-8.
29. Bakhtiyari M, Delpisheh A, Riahi SM, *et al.* Epidemiology of occupational accidents among Iranian insured workers. *Saf Sci*. 2012;50(7):1480-4.DOI: https://doi.org/10.1016/j.ssci.2012.01.015.
30. Davey S, Maheshwari C, Raghav SK, *et al.* Impact of occupational health hazards prevention messages on perceptions among rural clients in India: The outcomes of a panel study. *International Journal of Health System and Disaster Management*. 2017;5(1):11.
31. Kabir A, Farhana N, Akter F, *et al.* Sweeping practices, perceptions and knowledge about occupational safety and health hazards of street sweepers in Dhaka city, Bangladesh: a qualitative inquiry. *Int J Community Med Public Health*. 2015;2(3):237-43.

For peer review only

32. Budhathoki SS, Singh SB, Sagtani RA*, et al.* Awareness of occupational hazards and use of safety measures among welders: a cross-sectional study from eastern Nepal. *BMJ open*. 2014;4(6):e004646.
33. Prasad RV, Kanimozhy K, Konduru RK*, et al.* Occupational diseases and safety measures: Perceptions of saw mill workers–A qualitative study in Pondicherry, South India. *Indian Journal of Medical Specialities*. 2016;7(1):19-22.
34. Regmi PR, Van Teijlingen E, Mahato P*, et al.* The health of Nepali migrants in India: A qualitative study of lifestyles and risks. *Int J Environ Res Public Health*. 2019;16(19):3655.DOI: https://doi.org/10.3390/ijerph16193655.
35. Carter WS. Introducing occupational health in an emerging economy: a Nepal experience. *Ann Occup Hyg*. 2010;54(5):477-85.DOI: https://doi.org/10.1093/annhyg/meq036.
36. Joshi SK. Occupational Safety and Health in Nepal Revisited. *Int J Occup Saf Health* 2018;8(1):1-2.DOI: https://doi.org/10.3126/ijosh.v8i1.22921.
37. Zahoor H, Chan AP, Gao R*, et al.* The factors contributing to construction accidents in Pakistan. *Engineering, construction and architectural management*. 2017;24(3):463-85.DOI: https://doi.org/10.1108/ECAM-01-2016-0027.

For peer review only

Legends

Figure 1: Map of Nepal and Makwanpur district in Bagmati province *(source: https://nepalindata.com/)*

For peer review only

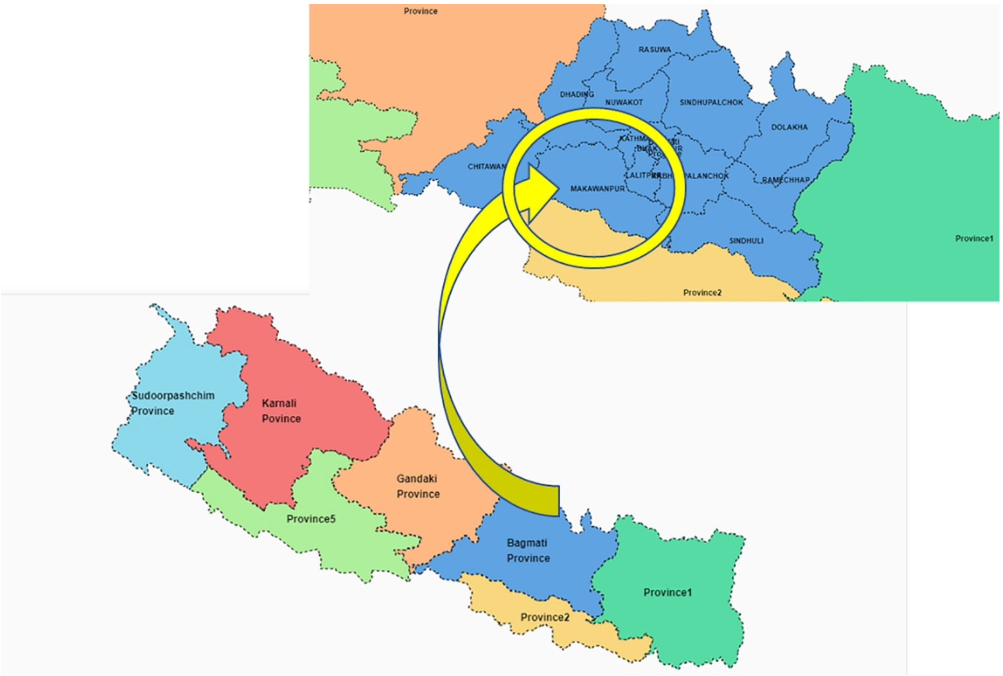

Figure 1: Map of Nepal and Makwanpur district in Bagmati province (source: https://nepalindata.com/)

188x125mm (300 x 300 DPI)

Supplementary materials

Supplementary file 1. A topic guide for the focus groups

INTRODUCTION

- Firstly, thank participants for joining discussion group
- Give a brief introduction of yourself, the purpose of the study
- Explain approximate time (audio- recording, handwritten notes)
- Encourage participants to speak clearly and only one at a time
- Explain that there will be no right or wrong views but in line with the topic
- Assure anonymity and confidentiality of participants and their views
- Explain how participants will not be identifiable from any record or report arising from the discussion
- Explain participant does not have to talk about anything they feel uncomfortable with and can stop at any time for any reason
- Sign consent form (check that all participants have signed that they are happy for the FG to be audio-recorded)

Warm-up
Introduction by participants with a brief background.

<u>**FOR REFERENCE IF DEFINITION NEEDED:**</u>

Injury definition: "The physical damage that results when a human body is subjected to energy that exceeds the threshold of physiological tolerance or results in lack of one or more vital elements, such as oxygen". All types of injury will be included in this study, including those described as intentional or unintentional. The terms intentional and unintentional denote whether an injury was meant to harm the victim or not. Intentional injuries include suicide and self-harm, homicide, assault and child abuse or purposeful neglect. Unintentional injury is any injury that originated suddenly, without any intent of self-harm, homicide or suicide. For example, falls, road traffic collisions, accidental poisoning, fire/burns, animal related injuries (bite, sting, crush or attack).

Operational definition:
<u>Home injury</u> - Any injury defined above that occurs in the home environment but not related to work. (Work is any means by which someone earns money. So, if someone works from home, e.g., making something to sell, growing food / animals to sell – that is work and that would be an occupational injury). If you farm to grow food just for home/family use, that could be a home injury.

<u>Occupational injury</u> - Any injury defined above that occurs during paid employment at any location, including those who might be working without a wage but works for the benefit of their family (e.g. agriculture and animal husbandry).

DISCUSSION/INTERVIEW
Now we shall begin our discussion in an organized way. For this please think about the injury that occurs in the home environment/work environment to any age of people who live or work in your community. Please start with an example you know about. In addition, please give us some examples that you have seen in your home/working area.

[To organize the group discussion, use the following questions to guide the discussion]:

Part 1. Perceptions of injuries
1.1 What do we mean by injury?
1.2 What injuries have you experienced / witnessed in the home/work environment (as appropriate)

Part 2. Risk factors for injuries

For peer review only - http://bmjopen.bmj.com/site/about/guidelines.xhtml

2.1 What caused those injuries in the home/work environment (as appropriate)? How did those injuries happen?

Part 3. Suggested intervention for injury prevention
3.1 What could be done (and by whom) to prevent other people having home/work injuries?
3.2 Home environment (as appropriate) - Whether the community people have had any change in their home environment and if so what that involved, and whether they think it helps? Also, do they have any safety equipment? If so, who provided it? Is it maintained? Does anyone check they are using it?
3.3 Work environment (as appropriate) - Whether the workers have had any safety training for their job and if so, what that involved and whether they think it helps? Also, do they have safety equipment? If so, who provides it? Is it maintained? Does anyone check they are using it?

Part 4. Barriers to and facilitators for such interventions
4.1 What might prevent such actions being done in the home/work environment (as appropriate)?
4.2 What might help such actions being done in the home/work environment (as appropriate)?

WRAP-UP

[At the end, there will be a short session for summarizing on a flip chart and recapping the whole discussion, thanking the participants and serving refreshments. This will be followed immediately by a meeting between the facilitator and note-taker]

Ending the discussion by saying: We have been discussing for about an hour. Do you think we missed anything that needs to be discussed?

FG discussion recording details

**Date:**
**Palika's name:**
**Ward No.:**
**Location (venue):**
**Type of Target Group:**
**Number of participants:**
**Age group:**
**Level of education:**
**Length of session:**
**Facilitator's name**
**Note taker' name:**
**Observation (if any):**
**Recorder No.:**

For peer review only - http://bmjopen.bmj.com/site/about/guidelines.xhtml

Supplementary file 2. A semi-structured interview schedule for key interviews

INTRODUCTION

Explain the study, ask if the participant has read the information sheet. Use the information sheet to explain the purpose of study, the voluntary nature of the interview, that the information they give will be kept confidential and that they can withdraw at any time.

Audio-recording: explain and ask for consent to use.

Consent: explain and ensure consent form is signed has a thumb print.

FOR REFERENCE IF DEFINITION NEEDED:

Injury definition: "The physical damage that results when a human body is subjected to energy that exceeds the threshold of physiological tolerance or results in lack of one or more vital elements, such as oxygen". All types of injury will be included in this study, including those described as intentional or unintentional. The terms intentional and unintentional denote whether an injury was meant to harm the victim or not. Intentional injuries include suicide and self-harm, homicide, assault and child abuse or purposeful neglect. Unintentional injury is any injury that originated suddenly, without any intent of self-harm, homicide or suicide. For example, falls, road traffic collisions, accidental poisoning, fire/burns, animal related injuries (bite, sting, crush or attack).

Operational definition:

Home injury: Any injury defined above that occur in the home environment (home and near environment) but not related to work. (Work is any means by which someone earns money. So, if someone works from home, e.g., making something to sell, growing food / animals to sell – that is work and that would be an occupational injury). If you farm to grow food just for home/family use, that could be a home injury.

Occupational injury: Any injury defined above that occurs during work at any location, including those who might be working without a wage but works for the benefit of their family (e.g. agriculture and animal husbandry).

Opening:

We would like to ask you a few questions about injuries, how they happen and what might prevent them. There are no right or wrong answers; we just want to find out what you think helps to keep people safe from accidents and what makes it hard to do this.

1. What is your understanding of what an injury is? Can you give an example, please?
   (if they are not correct, explain what we mean by the word 'injury')

2. What injuries have you experienced / witnessed in the home/work environment (as appropriate)? Can you describe one instance?

   (If they can't give an example, ask them to imagine a near miss or something that they could envisage happening.)

3. What do you think might have caused those injuries/why do you think they happened? How did those injuries happen? Can you describe the reasons, please?

   (Try and get them to give as much detail as possible. Repeat what they have said and ask them more details, if need be.)

4. Do you have any ideas about how injuries in the home/at work (as appropriate) could be prevented?
   a. What sort of thing might help?
   b. Who might do this?
   c. How might this be done?

For peer review only - http://bmjopen.bmj.com/site/about/guidelines.xhtml

5. Have you made any changes to your home/work to try and reduce the likelihood of an injury happening? Have you tried to make some changes?

    a. Has anyone in the community (home or work) made any changes? If yes, what? If yes, do you think it helped?
    b. Is any safety equipment used in the home/work at all? If yes, what and who provided it? Is it maintained (if relevant)? Does anyone check it (if relevant)?

    (They might not be in a position to make any changes so ask whether they have ideas for what could have been done and if they have made suggestions to anyone.)

6. **Work environment:** have the workers had any safety training for their job, this includes any training for use of safety equipment? Who provided it? Is this undertaken regularly? Who ensures that it is completed?

Barriers and facilitators to injury prevention

7. What might prevent or deter people trying to use an intervention to stop injuries (home or work)? Why might people not use something that will help to prevent an injury? Do you have any ideas?

    (Try to get some detail here about cost, practicality, someone taking ownership, leading the intervention etc.)

8. What might help or encourage people to use an intervention to stop injuries (home or work)? Do you have any ideas?

    (Try to get some detail here about cost, practicality, someone taking ownership, leading the intervention etc.)

9. Do you have any other comments or anything else that you would like to say?

Thank you very much for talking to me. We will use the information that you have given me to describe the hazards that cause injuries and the possible interventions that might be used to help prevent them.

KIIs recording details

**Date:**
**Palika's name:**
**Ward No.:**
**Location (venue):**
**Type of Target Group:**
**Age group:**
**Level of education:**
**Length of interview:**
**Interviewer's name:**
**Observation (if any):**
**Recorder No.:**
**Personal identification code**

Supplementary files

For peer review only - http://bmjopen.bmj.com/site/about/guidelines.xhtml

Perceptions of injury risk in the home and workplace in Nepal: a qualitative study

Consolidated criteria for reporting qualitative studies (COREQ): 32-item checklist (Tong, Sainsbury & Craig, 2007)

No. Item	Guide questions	Description/Page
**Domain 1: Research team and reflexivity**		
Personal Characteristics		
1. Interviewer/facilitator	Which author/s conducted the interview or focus group?	EJ facilitated 7 Focus groups and 8 interviews and SB facilitated 2 focus groups and 1 interview Page: 5, DA helped to organize focus groups and interviews
2. Credentials	What were the researcher's credentials? E.g. PhD, MD	EJ: Masters in Nursing<br>SB: PhD<br>TD: PhD<br>JM: PhD<br>SRM: MD<br>DA: Masters in Social Science<br>SKJ: PhD
3. Occupation	What was their occupation at the time of the study?	TD, EJ, SB, JM: Researchers<br>DA: Site Coordinator<br>SKJ: Researcher, Academician<br>SRM: Researcher, Pediatrician
4. Gender	Was the researcher male or female?	TD, EJ. JM: Female<br>SB, SRM, SKJ, DA: Male
5. Experience and training	What experience or training did the researcher have?	All authors have relevant research experience and training on study design, data collection and analysis
Relationship with participants		
6. Relationship established	Was a relationship established prior to study commencement?	No relationship was established prior to study.
7. Participant knowledge of the interviewer	What did the participants know about the researcher? e.g. personal goals, reasons for doing the research	The participants were briefed about research objectives using study information sheet. Page:4
8. Interviewer characteristics	What characteristics were reported about the inter viewer/facilitator? e.g. Bias, assumptions, reasons and interests in the research topic	Trained researchers with experience in conducting injury research undertook the data collection.
**Domain 2: study design**		
Theoretical framework		
9. Methodological orientation and Theory	What methodological orientation was stated to underpin the study? e.g. grounded theory, discourse analysis, ethnography, phenomenology, content analysis	Inductive thematic analysis was done and the rationale for using it has been justified in the method section. Page:5
Participant selection		
10. Sampling	How were participants selected? e.g. purposive, convenience, consecutive, snowball	Purposive sampling using pre-specified sampling framework. Page:4-5

For peer review only

Perceptions of injury risk in the home and workplace in Nepal: a qualitative study

Consolidated criteria for reporting qualitative studies (COREQ): 32-item checklist (Tong, Sainsbury & Craig, 2007)

11. Method of approach	How were participants approached? e.g. face-to-face, telephone, mail, email	Face to face interviews and focus groups were conducted. Page:3-4
12. Sample size	How many participants were in the study?	A total of 83 in nine focus groups and nine interviews in eighteen different sessions. Page:5
13. Non-participation	How many people refused to participate or dropped out? Reasons?	One participant refused to participate. No reason was given.
Setting		
14. Setting of data collection	Where was the data collected? e.g. home, clinic, workplace	The data were collected at home and workplace, whichever was convenient and feasible for the participants. Page: 4
15. Presence of non-participants	Was anyone else present besides the participants and researchers?	A local field staff took notes during the focus group discussions. The field staff were not familiar with the participants.
16. Description of sample	What are the important characteristics of the sample? e.g. demographic data, date	Sex, age, participants role, location Page:6-7
Data collection		
17. Interview guide	Were questions, prompts, guides provided by the authors? Was it pilot tested?	Topic guide and interview schedule were developed for focus groups and interviews, respectively. The topic guide and interview schedule were pre-tested. Page:5
18. Repeat interviews	Were repeat inter views carried out? If yes, how many?	No.
19. Audio/visual recording	Did the research use audio or visual recording to collect the data?	Audio recording of focus groups and interviews with participants consent Page:4-5
20. Field notes	Were field notes made during and/or after the interview or focus group?	Brief field and observation notes were made during focus groups.
21. Duration	What was the duration of the inter views or focus group?	The average focus groups were 37 minutes and interviews were 31 minutes. Page:6
22. Data saturation	Was data saturation discussed?	No. Not required as per the study aim. Page: 4
23. Transcripts returned	Were transcripts returned to participants for comment and/or correction?	The transcripts were not returned to participants.
**Domain 3: analysis and findings**		
Data analysis		
24. Number of data coders	How many data coders coded the data?	Two
25. Description of the coding tree	Did authors provide a description of the coding tree?	No. Can be made available if requested
26. Derivation of themes	Were themes identified in advance or	Identified from the data.

For peer review only

Perceptions of injury risk in the home and workplace in Nepal: a qualitative study

Consolidated criteria for reporting qualitative studies (COREQ): 32-item checklist (Tong, Sainsbury & Craig, 2007)

	derived from the data?	
27. Software	What software, if applicable, was used to manage the data?	Translation, transcription, and data analysis were done manually with the use of NVivo to manage the data.
28. Participant checking	Did participants provide feedback on the findings?	The participants didn't provide feedback on the findings.
Reporting		
29. Quotations presented	Were participant quotations presented to illustrate the themes/findings? Was each quotation identified? e.g. participant number	The quotes from participants were used and identified using a personal identification code to ensure anonymity. Page:7-11
30. Data and findings consistent	Was there consistency between the data presented and the findings?	There was consistency between the data presented and the findings.
31. Clarity of major themes	Were major themes clearly presented in the findings?	Yes. Major themes are described. Page: 7-11
32. Clarity of minor themes	Is there a description of diverse cases or discussion of minor themes?	We presented the major themes. Diversity of the participants are described. Page: 4 & 5

For peer review only

BMJ Open

Perceptions of injury risk in the home and workplace in Nepal: a qualitative study

Journal:	*BMJ Open*
Manuscript ID	bmjopen-2020-044273.R2
Article Type:	Original research
Date Submitted by the Author:	24-Feb-2021

Complete List of Authors:	Joshi, Elisha; Kathmandu Medical College and Teaching Hospital, Community Medicine<br>Bhatta, Santosh; University of the West of England, Faculty of Health and Applied Sciences<br>Deave, Toity; University of the West of England, Bristol, Faculty of Health and Applied Sciences<br>Mytton, Julie; University West of England, Faculty of Health and Applied Sciences<br>Adhikari, Dhruba; Mother and Infant Research Activities<br>Manandhar, Sunil Raja; Mother and Infant Research Activities<br>Joshi, Sunil Kumar; Kathmandu Medical College and Teaching Hospital, Community Medicine

**Primary Subject Heading**:	Qualitative research
Secondary Subject Heading:	Public health, Sociology, Health policy, Evidence based practice
Keywords:	QUALITATIVE RESEARCH, PUBLIC HEALTH, Health & safety < HEALTH SERVICES ADMINISTRATION & MANAGEMENT, PREVENTIVE MEDICINE, SOCIAL MEDICINE

SCHOLARONE™
Manuscripts

I, the Submitting Author has the right to grant and does grant on behalf of all authors of the Work (as defined in the below author licence), an exclusive licence and/or a non-exclusive licence for contributions from authors who are: i) UK Crown employees; ii) where BMJ has agreed a CC-BY licence shall apply, and/or iii) in accordance with the terms applicable for US Federal Government officers or employees acting as part of their official duties; on a worldwide, perpetual, irrevocable, royalty-free basis to BMJ Publishing Group Ltd ("BMJ") its licensees and where the relevant Journal is co-owned by BMJ to the co-owners of the Journal, to publish the Work in this journal and any other BMJ products and to exploit all rights, as set out in our licence.

The Submitting Author accepts and understands that any supply made under these terms is made by BMJ to the Submitting Author unless you are acting as an employee on behalf of your employer or a postgraduate student of an affiliated institution which is paying any applicable article publishing charge ("APC") for Open Access articles. Where the Submitting Author wishes to make the Work available on an Open Access basis (and intends to pay the relevant APC), the terms of reuse of such Open Access shall be governed by a Creative Commons licence – details of these licences and which Creative Commons licence will apply to this Work are set out in our licence referred to above.

Other than as permitted in any relevant BMJ Author's Self Archiving Policies, I confirm this Work has not been accepted for publication elsewhere, is not being considered for publication elsewhere and does not duplicate material already published. I confirm all authors consent to publication of this Work and authorise the granting of this licence.

For peer review only

Perceptions of injury risk in the home and workplace in Nepal: a qualitative study

Corresponding Author

1. Elisha Joshi*
Nepal Injury Research Centre, Kathmandu Medical College, Duwakot, Bhaktapur, Nepal
ejoshi03@gmail.com; https://orcid.org/0000-0002-3534-276X

Co-Authors

2. Santosh Bhatta*
Faculty of Health and Applied Sciences, University of the West of England, Bristol, UK
https://orcid.org/0000-0001-6393-2495

**Elisha Joshi and Santosh Bhatta have contributed equally*

3. Toity Deave
Faculty of Health and Applied Sciences, University of the West of England, Bristol, UK
https://orcid.org/0000-0001-9966-8948

4. Julie Mytton
Faculty of Health and Applied Sciences, University of the West of England, Bristol, UK
http://orcid.org/0000-0002-0306-4750

5. Dhruba Adhikari
Mother and Infant Research Activities (MIRA), Makwanpur, Nepal

6. Sunil Raja Manandhar
Kathmandu Medical College and Mother and Infant Research Activities (MIRA), Makwanpur, Nepal
https://orcid.org/0000-0002-6583-3973

7. Sunil Kumar Joshi
Department of Community Medicine, Kathmandu Medical College Public Limited, Kathmandu, Nepal
https://orcid.org/0000-0002-2704-5060

Word Count: 3986 (Max 4000 words)

For peer review only - http://bmjopen.bmj.com/site/about/guidelines.xhtml

ABSTRACT

Objective: Injuries are a global health problem. To develop context specific injury prevention interventions, one needs to understand population perceptions of home and workplace injuries. This study explored a range of views about the perception of injuries in a variety of settings and identified barriers and facilitators to injury prevention.

Design: Qualitative study: interviews and focus groups.

Setting: Three administrative areas: Hetauda sub-metropolitan city, Thaha municipality (urban) and Bakaiya rural municipality in Makwanpur, Nepal.

Participants: Nine focus groups (74 participants) and nine one-to-one interviews were completed; workers from diverse occupations, residents (slum, traditional or modern homes), and local government decision makers participated in the study between May and August 2019. The interviews and discussions were audio-recorded, transcribed verbatim, translated into English and analysed thematically.

Results: Six themes were developed: unsafe home and workplace environment; inadequate supervision and monitoring; perceptions that injuries are inevitable; safety takes low priority-financial and behavioural considerations; safety education and training; government-led safety programmes and enforcement. Key barriers to injury prevention were perceived to be lack of knowledge about injury risk and preventive measures at both a community level and in the workplace. Facilitators were community level educational programmes and health and safety training to employees and employers. Participants stressed the importance of the role of government in planning future injury prevention programmes in different environments.

Conclusions: This study highlighted that both home and workplace injuries are complex and multifactorial. Lack of knowledge about injury risks and preventive measures, at both a community level and in the workplace, were found to be common barriers to injury prevention; perceived to be mitigated by educational programmes. Together with previously published epidemiological evidence, the barriers and facilitators identified in this study offers useful basis to inform policy and practice.

Keywords: Perception, injury risk, home, workplace, qualitative, Nepal

Strengths and limitations of this study

- Participants from diverse home environments, different work settings and different socio-economic backgrounds yielded a breadth of views.
- This is the first study to have explored qualitatively the views of the public on perceptions of injury risks at home and at work in Nepal.
- The study is not able to provide perceptions about injury risks and preventive measures by injury type.

For peer review only - http://bmjopen.bmj.com/site/about/guidelines.xhtml

INTRODUCTION

Injuries are a global health problem, although being predictable and largely preventable.(1) According to the global estimate, nearly 4.5 million people died from injuries in 2017, with a rate of disability-adjusted life years (DALYs) of 3267 per 100,000.(2) Ninety-percent of injury-related deaths occur in low- and middle-income countries (LMICs) and, in Nepal, there has been an increase in injury-related deaths from 6.3% to 9.2% between 1990 and 2017.(3) Globally, road traffic injuries, falls, burns, poisonings and suicides are the leading causes for unintentional and intentional injuries.(4) According to the International Labour Organization, more than 2.78 million deaths per year are estimated to be due to occupational injuries or workplace disease.(5) In Nepal, 200 workers die and 20,000 workers suffer from workplace injuries yearly.(6)

Recent evidence for injuries occurring at home,(7) identified parental supervision and teaching children about injury risks were as facilitators, while barriers to child injury prevention were identified as parents' lack of anticipation of injury risks and perceiving injuries as inevitable events.(8) Culturally acceptable prevention measures, appropriate supervision arrangements, separation of hazards and training children and parents about safety were suggested by a study of community perceptions in Makwanpur, Nepal.(9) Two community-based studies conducted in rural Nepal emphasised that unintentional child injuries were thought to be due to coincidence, bad luck, witchcraft or ill fate.(9, 10) Likewise, in Bangladesh, child drowning was believed to be a result of ill fate and unpreventable.(11) Rarely were the environmental and infrastructural factors thought by parents to be the cause of child injury.(9)

Workplace injuries are becoming a public health concern in all LMICs. One qualitative study undertaken in Bangladesh found that poor people were at greater risk of injury, employers were reluctant to take responsibility for workers and subcontracting workers was observed to increase the injury risk.(12) Despite the high level of awareness about the use of personal protective equipment (PPE) among Nepali workers, there was poor practice of using PPE.(13) A qualitative study conducted among Nepali migrant workers suggested that workplace injuries were due to lack of health and safety regulations, the risk-taking behaviour of workers and perceived work pressure.(14)

Human behaviour, being a complex phenomenon, is determined by environmental factors (such as social support/barriers, ability to change one's own environment), behavioural factors (such as skills, practice and self-efficacy) and personal factors (such as knowledge and, perception).(15) To understand health and safety behaviours, one needs

to understand how people perceive injury risks and what the factors are that influence their behaviours.(13) Little is known about how the people of Nepal perceive and deal with home and

For peer review only - http://bmjopen.bmj.com/site/about/guidelines.xhtml

workplace injuries or their risk factors. This study explored a range of views about the perception of injuries in a variety of settings and identified barriers and facilitators to injury prevention.

METHODS

Study design

We adopted a qualitative research methodology using focus group discussions and key informant interviews.

Study setting and participants

The study took place in Makwanpur district of Nepal (See Figure 1) that has a mixed topography similar to other districts in the country. The three administrative areas (‘*palikas*’) were selected purposively: Hetauda sub-metropolitan city (urban area), Thaha municipality (suburban area) and Bakaiya municipality (rural area). To ensure diversity of location, occupation, housing type and key government personnel, and to achieve the information power necessary to answer the research question,(16) the research team prepared a pre-specified sampling framework where we identified and listed key groups we wished to include. Members of the research team consulted with the existing networks and local government officers to identify the knowledgeable and experienced individuals and groups of participants. In each study area, a local non-governmental organisation (NGO), Mother and Infant Research Activities (MIRA), who have over 20 years of experience of working in Makwanpur district, Nepal, helped to identify prospective participants. The participants who met the inclusion criteria, were 18 years old or over and resident and/or working in one of the three *palikas*, were approached by the research team. Identified individuals were invited to take part, were given a participant information sheet and consent form. Consent forms were completed for those who agreed to take part. The research team conducted all the focus groups and interviews at a location and time convenient to the participant groups and individuals in each study site. There was no adverse consequence for non-participation and as a token of appreciation for their time and participation, each participant was given 1000 Nepali Rupees (equivalent to approximately 6 British Pounds).

For peer review only - http://bmjopen.bmj.com/site/about/guidelines.xhtml

Data collection

The focus groups (FGs) and interviews were conducted to explore views and issues of people residing in varied accommodation and environments and workers in their different workplaces. Interviews were conducted with key people, based on their role and nature of their job.

A topic guide for the focus groups and a semi-structured interview schedule were developed (see supplementary files). These were developed based on previous work undertaken by the research team, pretested and finalised prior to data collection. All focus groups and interviews were conducted face-to-face by either SB or EJ. With consent, the focus groups and interviews were digitally audio-recorded and written notes were taken concurrently by a note taker to capture non-verbal communication. The interview schedule and topic guide asked questions about, for example, perception of injuries, risk factors and barriers and facilitators to injury prevention.

Data management

Recordings were transcribed verbatim in Nepali and then translated into English with an aim to retain the original meaning of the statements. The transcriptions and translations were completed by experienced Mother and Infant Research Activities (MIRA) staff and verified by researchers (EJ & SB). Personal identifiable information from participants were removed from transcriptions and replaced with a unique identification code.

Data analysis

The interviews and focus groups data were analysed using inductive thematic analysis to search for repeated patterns of meaning or themes in the data.(17) NVivo qualitative data analysis software, Version 12, was used to arrange the codes systematically and collate data relevant to each code.(18) Two transcripts were coded by both EJ and SB and discussed with a third researcher (TD) to agree an initial coding framework which was then applied consistently across the transcripts. After coding all transcripts, the individual codes were reviewed and placed into clusters based on their similarities. The clusters were organised to develop candidate themes and barriers and facilitators were identified. Any discrepancies and differences in codes and themes developed were discussed with a third researcher (TD) and final themes were agreed.

Patient and Public Involvement

We worked with a local NGO (MIRA) and local government officials to identify relevant groups and key personnel to recruit as participants in our study. No patients were involved in this study.

For peer review only - http://bmjopen.bmj.com/site/about/guidelines.xhtml

RESULTS

Participants' characteristics

We conducted nine focus groups and nine one-to-one interviews with a total of 83 participants across three municipalities. Out of ten participants that we approached, one participant refused to participate in the interview, hence participation was 90% for the interviews. The participation for the focus groups was 100%. The average length of each focus group was 37 minutes and interview was 31 minutes. A total of six focus groups and six interviews were conducted in Hetauda sub-metropolitan city, two focus groups and two interviews in Bakaiya rural municipality and one focus group and one interview in Thaha municipality. Participants in the focus groups were from similar backgrounds (table 1) whereas interviewees were representatives from different institutes and organisations (table 2).

Table 1. Characteristics of the focus group participants

Focus Group ID	Participants	Participant numbers	Age range of participants (years)	Gender
FG 1	Residents in rural, traditionally built home	8	21-55	M:4 F:4
FG 2	Residents in rural, concrete/modern home	9	21-55	M:5 F:4
FG 3	Residents in urban area	8	35-50	M:4 F:4
FG 4	Haulage/truck drivers	9	40-57	M:9
FG 5	Residents in slum area	8	18-59	M:4 F:4
FG 6	Skilled construction workers: plumber, welder, electrician	8	21-50	M:8
FG 7	Farmers (agriculture, animal husbandry)	8	37-51	M:4 F:4
FG 8	Factory workers (cement factory)	9	26-41	M:8 F:1
FG 9	Trade professionals	7	30-53	M:7

Male: M, Female: F; FG: Focus Group;

Table 2. Characteristics of the interview participants

Interviewee ID	Participants	Age range of participants (years)	Gender
KII 1	President of mothers' group	40-50	F
KII 2	Consultant in charge of hospital emergency department	20-30	F
KII 3	Healthcare Manager of municipality	40-50	M
KII 4	Healthcare Manager of sub-metropolitan city	40-50	F
KII 5	Trade union representatives	50-60	M

For peer review only - http://bmjopen.bmj.com/site/about/guidelines.xhtml

KII 6	Senior Officer of Hetauda industrial area	40-50	M
KII 7	Representative of transport workers	40-50	M
KII 8	Rural municipality officer	40-50	M
KII 9	Senior executive of education	50-60	M

KII: Key Informant Interview

Themes

Six themes were identified from the focus groups and interview data and these are described below. Relevant quotes are presented to illustrate the key themes.

1. Unsafe home and workplace environment

The physical infrastructure and home environment were perceived as common causes of unintentional injuries at home. The participants said that the lack of railings on stairs was the leading cause of fall injuries among people living in traditionally built rural houses, especially hazardous to children. Participants from urban areas raised their concerns that elderly people sustained fall injuries due to slipping on marble or tiled bathroom floors. Burn injuries were common among children and women where firewood was used for cooking.

> *While the kids are running in the balcony, they jump from there and get hurt. It's like that in these mud houses...there is no fence[railing]!" (FGD_1, P8)*

Regarding workplace injuries, different employees, like factory workers, healthcare staff and farmers all highlighted that falls, cuts and burns were common injuries. Some described how they have to work in risky environments sometimes without safety equipment. One factory worker reported:

> *I have seen workers suffering from the pain due to heat. They have to take out rods from 300 degrees of heat by bare hands. They even don't wear gloves and boots. They use scissors [instead of tongs] ….* (KII 6)

Many participants explained that workers who are illiterate and suffer from poverty were those who worked under private contractors who are less likely to provide safety equipment.

2. Inadequate supervision and monitoring

The participants highlighted the need for close supervision and monitoring of children and teenagers. One of the focus groups reported that there had been incidents of poisoning when children find liquids that look like food or drink. Although parents said they were aware of this, they said that careful supervision was difficult for working and single parents and, therefore, injuries occurred.

For peer review only - http://bmjopen.bmj.com/site/about/guidelines.xhtml

The parents have to work throughout the day to earn food and the children are guided either by the school or without anyone proper guidance. The parents are unknown about their children's behaviour. They simply grow without their parents' guidance... (KII 9)

3. Perception that injuries are inevitable

In regards to both home and workplace injuries, participants highlighted that injuries occur by chance, unexpectedly and are normal in their situation. Some participants felt that one's luck is responsible for occurrence of injuries. Acceptance of injuries as a normal part of life was a predominant finding; they were uncertain about how injuries could be prevented. They believed that such injuries that occurred abruptly could not be prevented, despite them being careful when performing tasks.

I feel that people get injured if they have misfortune or if the movement of planets that influences the destinies of people is not good or else, they [people] won't get injured. Even if we are careful, we might get injured due to our bad fortune. (FG 5, P2)

It's like this. In the workshops the cuts are normal. We need to play with the metals. While doing that [playing with metals] the sharp edges of it [metals] can give cuts to us. We also get normal cuts when we are cutting down the pipes. (FGD_6_P2)

4. Safety takes low priority: financial and behavioural considerations

Most participants said that when they were at home they didn't think about safety. Carelessness when completing tasks in a hurry was the most common reason for injuries occurring in the home and workplace.

Most of the injuries take place due to our carelessness. Like we throw nails and other sharp materials here and there…. Another reason is … performing any kind of activities hurriedly while walking … (FG 2, P2)

Participants from rural villages confessed that financial constraints hindered them from implementing safety measures like installing railings to balconies as they struggled to meet their families' basic needs.

Due to this economic barrier, we are unable to manage everything… (FG 1, P5)

Participants described the common practice of employers who pressurised their workers to work fast without providing PPE. Inadequate capital was one reason for employers for not providing PPE or installing safer, automatic machines.

For peer review only - http://bmjopen.bmj.com/site/about/guidelines.xhtml

> *...the manufacturers of construction factories like grill factories are more concerned about how to increase their production leading the workers to work fast and carelessness at work.* (KII 5)

The workers stated that, not only did they have to work without PPE, especially the contracted workers, but most of them worked carelessly, misusing or not using safety equipment even when the PPE was available. Overconfidence and feeling that nothing would happen to them led the workers to take such risks.

> *Even if we have safety equipment, we get careless and do not use them thinking that the work we are about to perform just takes 2 minutes of time and we won't require it [safety equipment]. But, in that 2 minutes of time any incident may occur.* (FG 7, P3)

Senior personnel emphasized that it was the role of supervisors to consider safety, raise awareness and orient the workers to the dangerous aspects of their work.

> *What I would say is once you recruit the person, he/she should be taught everything. Every industry should manage this and conduct orientation. The industry should make employees learn about the culture of work. But this culture isn't practised.* (KII 6)

5. Safety Education and Training

Additional to the behavioural and financial aspects, participants reported that lack of awareness was a major contributing factor that led to home and workplace injuries; illiteracy and a lack of understanding about injury was prevalent. Almost all the participants voiced the need for an awareness programme, that it would be a crucial enabling factor for injury prevention, both in the home and workplace.

> *In case of home, there should be awareness program because they [community people] don't know. They should be educated such that they can bring change in their behaviour. It is because they don't understand.* (KII 3)

Regarding home injury, many participants stated that a programme to reduce injuries at home should be conducted by local community groups and their leaders, such as the mothers' groups. Participants believed that effectiveness of programmes relied on being led by injury experts in collaboration with NGOs, International Non- Governmental Organisations (INGOs) and local government.

> *If the outsiders go and tell them [community people] they might listen. But if we locals tell them they won't listen to us.* (FG2, P4)

For peer review only - http://bmjopen.bmj.com/site/about/guidelines.xhtml

Participants also felt that such injury prevention programmes should be delivered to school children, teachers, parents and community leaders, using social media or community mobilisation.

> *For that we must gather all the family members in a place and discuss about this matter [injuries]. We must discuss about how injuries take place and how we can reduce it [injuries]....* (FG 3, P7)

Factory workers raised concerns about the lack of institute to train workers, to make them aware of occupational health and safety. Skilled workers and laborers highlighted the need for safety training, that it could be provided and monitored by industries, NGOs and the Ministry of Labour. There was a consensus among factory workers that training needed to be inclusive of all workers, company and contract staff, including the employers, that it should be done regularly, addressing any technological updates and human tendency to forget. Pamphlets or brochures with brief messages would raise awareness amongst the workers.

> *The main important thing is to [make aware] the employer, those who employ the workers. Basically, they should be aware of the health of the workers. We should also be able to make the workers understand that, if you are healthy then you can do lot of work...* (KII 5)

6. Government led safety programmes and enforcement

Almost all participants thought that the government's role was pivotal for injury prevention activities. They thought that government lacked focus on injury prevention activities due to the lack of injury data. Most participants believed that the local government authorities needed to take a lead on injury prevention activities. On probing what would work, they thought that with NGOs, INGOs and injury experts collaborating, strategic planning could be achieved. Participants provided examples of how the municipality was working in partnership with some organisations for community health development and women empowerment. For example, the municipality was working with research organisations to prioritise injury prevention programmes.

> *The local government along with the NGO/INGOs have mobilized the mother's group... The local government should launch such a program [injury prevention] which will enhance them [mothers] and help to promote the injury related activities.* (KII 9)

The participants highlighted the role of central government could be to formulate uniform policies, with strict enforcement of rules and regulations to prevent injuries both in the home and workplace. Periodic monitoring visits to industries undertaken by the government and private sectors was felt to be mandatory to ensure safety standards were being followed.

For peer review only - http://bmjopen.bmj.com/site/about/guidelines.xhtml

The government of Nepal should formulate standard protocol to prevent injuries at industries. If there isn't environment as mentioned in the protocol then they shouldn't be renewed and the industry should be closed immediately. (KII 3)

Barriers and facilitators to injury prevention

The key barrier to injury prevention identified by participants was the lack of knowledge about injury risk and preventive measures, at both a community level and in the workplace. Other barriers included: safety not being a priority and the inability to improve the safety of the home or working environments due to financial constraints. The barriers, particularly for child injury prevention, included the inability of parents to provide adequate supervision. Participants identified that safety in the home and workplace could be improved at a community level, through educational programmes to raise awareness about how injuries could be prevented. In the workplace, health and safety training could be provided for employers and employees and safety laws introduced with rigorous enforcement. The involvement of external agencies and involvement of government authorities could facilitate the implementation and effectiveness of such programmes, training and legislation.

DISCUSSION

This study found that injuries were perceived as a problematic issue and preventive measures have been neglected across different settings.

Home injuries

In our study, the home environment and socioeconomic aspects were highlighted as the main contributing factors for injuries such as falls, burns and poisonings. Unsafe buildings and cooking on open fires have been identified previously as injury hazards(19) and, in a countrywide population-based study conducted in Nepal, falls were found to be the leading cause of injury.(20) A household survey that assessed home hazards for child injury in rural Nepal, found that 98% of households did not have protective railings on stairs, more than 80% households had no window bars and 50% households lacked a protective barrier on their balconies.(21) Similar to the findings from Bangladesh, the study participants perceived their financial constraints to be one of the barriers to prevention of home injuries.(22)

The findings from our study supports those from previous epidemiological evidence where children were found to be at high risk of burns and that the home was hazardous.(23, 24) Participants in our study provided the context and reported reasons for some of the more common injuries identified

For peer review only - http://bmjopen.bmj.com/site/about/guidelines.xhtml

by other studies. For example, open fires for cooking was unsafe(25) and the majority of households (61%) in rural areas of Nepal had chemicals or fertilisers within the reach of children.(21)

Some parents believed that injuries were a normal part of child development, bad luck, witchcraft or ill fate. This fatalism, in relation to injury, has been reported in previous studies conducted in LMICs(7, 26, 27) but no method to address this belief has been developed. A qualitative study in Nepal showed that when people are unable to rationalize the cause then the concept of fate is ascribed.(28) This lack of knowledge in the community has been reported in previous studies conducted in Nepal.(9, 10) In line with previous studies, the participants of this study also highlighted the need for interventions at all levels, such as government, NGOs and the local community.(22)

Workplace injuries

Minor injuries were reported as being common occurrences within the workplace but fatal injuries and those that led to long-term disability were also reported. Study participants believed that poverty and illiteracy were interlinked and were the root cause of workplace injuries. This might be because employers tended to hire the poorer workers who are ready to take on risky jobs but with a relatively higher wage(12) or that, similar to our study, carelessness was a significant causal factor, as found in one study of Iranian workers.(29)

Study participants stressed the need to raise awareness amongst workers about the importance of PPE use via regular and periodic occupational health and safety education programmes. A study in India demonstrated that the understanding of occupational hazards was significantly associated with the literacy status of workers and delivering occupational health education programmes augmented the understanding of Occupational Health and Safety (OHS).(30) Another study suggested that such programmes should be delivered together with behaviour change counselling(31) but our study participants believed that awareness programmes, with adequate PPE and enforcement of occupational standards, would suffice for adopting safe workplace practices. One study found that high levels of awareness of occupational hazards and use of PPE did not lead to the actual use of PPE.(32) Further exploration is needed about why workers don’t use PPE despite the availability and training. Unlike other studies, which highlighted lack of user-friendly PPE and ineffective PPE(33, 34), our study found both an absence of formal training prior to commencing work and lack of PPE which created environments conducive to injuries.

For peer review only

For peer review only - http://bmjopen.bmj.com/site/about/guidelines.xhtml

In Nepal, the 1992 Occupational Health and Safety law did not specify how to evaluate or enforce the law.(35) After the revision in 2017, it explicitly described the roles of employees and employers in adhering to OHS standards, yet legislation is silent on the supervision and monitoring of the implementation status.(36) In line with other studies, study participants raised concerns about poor enforcement of safety rules and regulations by government agencies.(37) Whilst the participants felt that this was needed, they also felt that neither party prioritised OHS; this was felt to be due to the lack of inspection and lack of enforcing penalty or fines for infringing the rules. This study highlights the need for a co-ordinated approach to injury prevention. Through the unified efforts of government, NGOs, communities and other professionals, social inequalities, enforcement issues and educational activities could be addressed and delivered.

Strengths and limitations

To the best of our knowledge, this is the first qualitative study that has reported information about home and workplace injuries among people living in varied settings and involved in various occupations in Nepal, Makwanpur district. The study design ensured a diversity of perspectives about both home and workplace injuries from people living in different home environments, those working in different occupations, from different levels of authority and from different socio-economic backgrounds. A limitation of the study is that it does not provide information on injury specific interventions but it does present a sound basis from which injury researchers can explore specific injury risks and identify measures to remove them.

Future Directions and Possible solutions

Based on participants' suggestions, home-based visits and awareness campaigns could be potential interventions for home injury prevention. Regarding prevention of workplace injuries, our study found that there was a need for regular workplace training for both employees and employers on safety measures, along with a mechanism to ensure levels of knowledge were maintained. Our study also stressed the need for a cultural shift so that greater self-efficacy to keep oneself and ones family and work colleagues safe becomes the norm. Findings from our study, collated with those of epidemiological studies, will be a good starting point to inform policy-level discussions. This would enable formulation and enforcement of policies and strategies related to home and workplace injury prevention.

CONCLUSION

The findings of this study highlighted that both home and workplace injuries are complex and multifactorial and are influenced by personal, situational and environmental factors. Most

For peer review only - http://bmjopen.bmj.com/site/about/guidelines.xhtml

importantly, lack of knowledge about injury risks and preventive measures, at both a community level and in the workplace were found to be common barriers to injury prevention. The introduction and implementation of educational home safety programmes delivered within the community and as an occupational safety programme within the workplace would be welcomed. Together with previously published epidemiological evidence, the perceptions of risk and perceived barriers and facilitators to safety identified in this study provide a useful basis on which policy makers can establish their decision-making when addressing home and workplace injuries in Nepal.

Footnotes

Author affiliations

1. Nepal Injury Research Centre, Kathmandu Medical College Public Limited, Kathmandu, Nepal
2. Faculty of Health and Applied Sciences, University of the West of England, Bristol, UK
3. Mother and Infant Research Activities, Kathmandu, Nepal
4. Department of Community Medicine, Kathmandu Medical College Public Limited, Kathmandu, Nepal

Acknowledgement:

We acknowledge the support of all three Palika authorities for approving the conduct of this study. We are grateful to all the staff at Mother and Infant Research Activities who lent their 20 years of research field experience to our study, supporting recruitment of the study participants and providing the logistics to conduct data collection. We are thankful to all the participants of our study who consented to participate and share their experience. We would like to acknowledge the support of the wider research team in the NIHR Global Health Research Group at the Nepal Injury Research UK (especially Emer Brangan) and Nepal team and administrative support of Kathmandu Medical college who have supported the study.

Contributors:

SB, TD, JM, SKJ contributed to the conception and design of the study. SB drafted the protocol design, methods and data analysis plan. SB and EJ contributed to data collection with support from SRM, DA. SB and EJ led the analysis, interpretation of data and drafted the manuscript. EJ and SB drafted and finalised the manuscript with equal contributions. All the authors contributed to drafts and approved the final manuscript.

Funding:

For peer review only

This research was funded by the National Institute for Health Research (NIHR) (Ref:16/137/49) using UK aid from the UK Government to support global health research. The views expressed are those of the author(s) and not necessarily those of the NIHR or the UK Department of Health and Social Care.

Competing interest: None declared

Patient Consent for Publication: Not required

Data Sharing Statement: Data sets are de-identified transcripts of nine interviews and nine focus groups (in Nepali translated in English). Data are available on request from corresponding author.

Ethics approval: Ethical approval was obtained from the Nepal Health Research Council (Reg. no. 779/2018) and the University of the West of England, Bristol, Faculty Research Ethics Committee (Reference: HAS.19.04.157). Institutional approval from each municipality office was also obtained for conducting the study in that location.

REFERENCES

1. World Health Organization. Injuries and violence: the facts 2014. Geneva, Switzerland; 2014.
2. James SL, Castle CD, Dingels ZV*, et al.* Global injury morbidity and mortality from 1990 to 2017: results from the Global Burden of Disease Study 2017. *Inj Prev*. 2020.DOI: http://dx.doi.org/10.1136/injuryprev-2019-043494.
3. Pant PR, Banstola A, Bhatta S*, et al.* Burden of injuries in Nepal, 1990–2017: findings from the Global Burden of Disease Study 2017. *Inj Prev*. 2020;0:1-10.DOI: http://dx.doi.org/10.1136/injuryprev-2019-043309.
4. Mock CN, Smith KR, Kobusingye O*, et al.* Injury prevention and environmental health: key messages from Disease Control Priorities. 2017.
5. Safety and health at work [Internet]. Internatioanl Labour Organization(ILO). 2020 [Accessed July 7 2020]. Available from: https://www.ilo.org/global/topics/safety-and-health-at-work/home/lang--en/index.htm.
6. Joshi SK, Shrestha S, Vaidya S. Occupational safety and health studies in Nepal. *Int J Occup Saf Health*. 2011;1(1):19-26.DOI: https://doi.org/10.3126/ijosh.v1i1.4725.
7. Butchart A, Kruger J, Lekoba R. Perceptions of injury causes and solutions in a Johannesburg township: implications for prevention. *Soc Sci Med*. 2000;50(3):331-44.DOI: https://doi.org/10.1016/S0277-9536(99)00272-5.
8. Ablewhite J, Peel I, McDaid L*, et al.* Parental perceptions of barriers and facilitators to preventing child unintentional injuries within the home: a qualitative study. *BMC Public Health*. 2015;15(1):280.
9. Pant PR, Towner E, Pilkington P*, et al.* Community perceptions of unintentional child injuries in Makwanpur district of Nepal: a qualitative study. *BMC public health*. 2014;14(1):476.
10. Bhatta S. Community-based home injury risk assessment in rural Nepal(Thesis). UK: University of the West of England; 2017.
11. Rahman A, Shafinaz S, Linnan M*, et al.* Community perception of childhood drowning and its prevention measures in rural Bangladesh: A qualitative study. *Aust J Rural Health*. 2008;16(3):176-80.DOI: https://doi.org/10.1111/j.1440-1584.2008.00969.x.
12. Akram O. Occupational health, safety and extreme poverty: a qualitative perspective from Bangladesh. *Int J Occup Saf Health*. 2014;4(1):41-50.DOI: https://doi.org/10.3126/ijosh.v4i1.10654.

For peer review only - http://bmjopen.bmj.com/site/about/guidelines.xhtml

13. Sapkota S, Lee A, Karki J, *et al.* Risks and risk mitigation in waste-work: a qualitative study of informal waste workers in Nepal. *Public Health in Practice*. 2020;1:100028.DOI: https://doi.org/10.1016/j.puhip.2020.100028.
14. Adhikary P, Keen S, Van Teijlingen E. Workplace accidents among Nepali male workers in the Middle East and Malaysia: A qualitative study. *J Immigr Minor Health*. 2019;21(5):1115-22.DOI: https://doi.org/10.1007/s10903-018-0801-y.
15. Gibson SK. Social learning (cognitive) theory and implications for human resource development. *Advances in developing human resources*. 2004;6(2):193-210.
16. Malterud K, Siersma VD, Guassora AD. Sample size in qualitative interview studies: guided by information power. *Qual Health Res*. 2016;26(13):1753-60.DOI: https://doi.org/10.1177%2F1049732315617444.
17. Braun V, Clarke V. *Successful qualitative research: A practical guide for beginners*: sage; 2013.
18. Maher C, Hadfield M, Hutchings M, *et al.* Ensuring rigor in qualitative data analysis: A design research approach to coding combining NVivo with traditional material methods. *Int J Qual Methods*. 2018;17(1):1609406918786362.DOI: https://doi.org/10.1177%2F1609406918786362.
19. Alonge O, Agrawal P, Talab A, *et al.* Fatal and non-fatal injury outcomes: results from a purposively sampled census of seven rural subdistricts in Bangladesh. *Lancet Glob Health*. 2017;5(8):e818-e27.DOI: https://doi.org/10.1016/S2214-109X(17)30244-9.
20. Gupta S, Gupta SK, Devkota S, *et al.* Fall injuries in Nepal: a countrywide population-based survey. *Ann Glob Health*. 2015;81(4):487-94.DOI: https://doi.org/10.1016/j.aogh.2015.07.004.
21. Bhatta S, Mytton JA, Deave T. Assessment of home hazards for non-fatal childhood injuries in rural Nepal: a community survey. *Inj Prev*. 2020.DOI: http://dx.doi.org/10.1136/injuryprev-2019-043482.
22. Jagnoor J, Keay L, Jaswal N, *et al.* A qualitative study on the perceptions of preventing falls as a health priority among older people in Northern India. *Inj Prev*. 2014;20(1):29-34.DOI: http://dx.doi.org/10.1136/injuryprev-2012-040707.
23. Khandarmaa T-O, Harun-Or-Rashid M, Sakamoto J. Risk factors of burns among children in Mongolia. *Burns*. 2012;38(5):751-7.DOI: https://doi.org/10.1016/j.burns.2011.11.006.
24. Rybarczyk MM, Schafer JM, Elm CM, *et al.* A systematic review of burn injuries in low-and middle-income countries: epidemiology in the WHO-defined African Region. *Afr J Emerg Med*. 2017;7(1):30-7.DOI: https://doi.org/10.1016/j.afjem.2017.01.006.
25. He S, Alonge O, Agrawal P, *et al.* Epidemiology of burns in rural Bangladesh: An update. *Int J Environ Res Public Health*. 2017;14(4):381.DOI: https://doi.org/10.3390/ijerph14040381.
26. Beard MR, Watson MC, Clifford MJ. Parents' perceptions of unintentional paediatric burn injuries—A qualitative study. *Burns*. 2020.DOI: https://doi.org/10.1016/j.burns.2019.12.009.
27. Munro SA, Van Niekerk A, Seedat M. Childhood unintentional injuries: the perceived impact of the environment, lack of supervision and child characteristics. *Child Care Health Dev*. 2006;32(3):269-79.DOI: https://doi.org/10.1111/j.1365-2214.2006.00593.x.
28. Hagaman AK, Khadka S, Wutich A, *et al.* Suicide in Nepal: Qualitative findings from a modified case-series psychological autopsy investigation of suicide deaths. *Cult Med Psychiatry*. 2018;42(3):704-34.DOI: https://doi.org/10.1007/s11013-018-9585-8.
29. Bakhtiyari M, Delpisheh A, Riahi SM, *et al.* Epidemiology of occupational accidents among Iranian insured workers. *Saf Sci*. 2012;50(7):1480-4.DOI: https://doi.org/10.1016/j.ssci.2012.01.015.
30. Davey S, Maheshwari C, Raghav SK, *et al.* Impact of occupational health hazards prevention messages on perceptions among rural clients in India: The outcomes of a panel study. *International Journal of Health System and Disaster Management*. 2017;5(1):11.
31. Kabir A, Farhana N, Akter F, *et al.* Sweeping practices, perceptions and knowledge about occupational safety and health hazards of street sweepers in Dhaka city, Bangladesh: a qualitative inquiry. *Int J Community Med Public Health*. 2015;2(3):237-43.

For peer review only

32. Budhathoki SS, Singh SB, Sagtani RA*, et al.* Awareness of occupational hazards and use of safety measures among welders: a cross-sectional study from eastern Nepal. *BMJ open*. 2014;4(6):e004646.
33. Prasad RV, Kanimozhy K, Konduru RK*, et al.* Occupational diseases and safety measures: Perceptions of saw mill workers–A qualitative study in Pondicherry, South India. *Indian Journal of Medical Specialities*. 2016;7(1):19-22.
34. Regmi PR, Van Teijlingen E, Mahato P*, et al.* The health of Nepali migrants in India: A qualitative study of lifestyles and risks. *Int J Environ Res Public Health*. 2019;16(19):3655.DOI: https://doi.org/10.3390/ijerph16193655.
35. Carter WS. Introducing occupational health in an emerging economy: a Nepal experience. *Ann Occup Hyg*. 2010;54(5):477-85.DOI: https://doi.org/10.1093/annhyg/meq036.
36. Joshi SK. Occupational Safety and Health in Nepal Revisited. *Int J Occup Saf Health* 2018;8(1):1-2.DOI: https://doi.org/10.3126/ijosh.v8i1.22921.
37. Zahoor H, Chan AP, Gao R*, et al.* The factors contributing to construction accidents in Pakistan. *Engineering, construction and architectural management*. 2017;24(3):463-85.DOI: https://doi.org/10.1108/ECAM-01-2016-0027.

For peer review only

Legends

Figure 1: Map of Nepal and Makwanpur district in Bagmati province *(source: https://nepalindata.com/)*

For peer review only

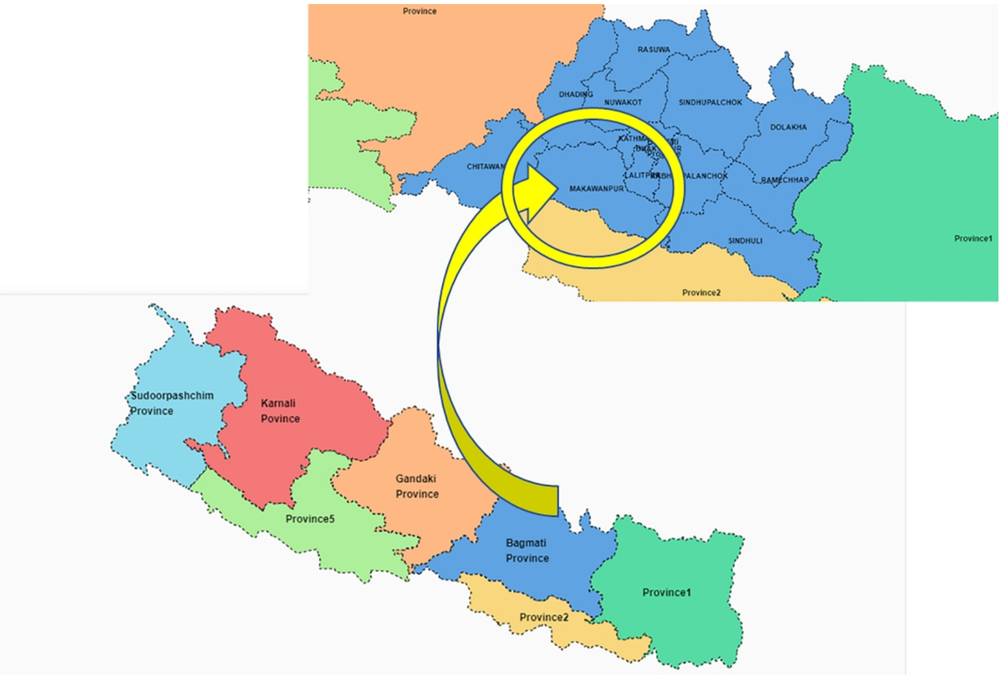

Figure 1: Map of Nepal and Makwanpur district in Bagmati province (source: https://nepalindata.com/)

188x125mm (300 x 300 DPI)

Supplementary materials

Supplementary file 1. A topic guide for the focus groups

INTRODUCTION

- Firstly, thank participants for joining discussion group
- Give a brief introduction of yourself, the purpose of the study
- Explain approximate time (audio- recording, handwritten notes)
- Encourage participants to speak clearly and only one at a time
- Explain that there will be no right or wrong views but in line with the topic
- Assure anonymity and confidentiality of participants and their views
- Explain how participants will not be identifiable from any record or report arising from the discussion
- Explain participant does not have to talk about anything they feel uncomfortable with and can stop at any time for any reason
- Sign consent form (check that all participants have signed that they are happy for the FG to be audio-recorded)

Warm-up
Introduction by participants with a brief background.

FOR REFERENCE IF DEFINITION NEEDED:

Injury definition: "The physical damage that results when a human body is subjected to energy that exceeds the threshold of physiological tolerance or results in lack of one or more vital elements, such as oxygen". All types of injury will be included in this study, including those described as intentional or unintentional. The terms intentional and unintentional denote whether an injury was meant to harm the victim or not. Intentional injuries include suicide and self-harm, homicide, assault and child abuse or purposeful neglect. Unintentional injury is any injury that originated suddenly, without any intent of self-harm, homicide or suicide. For example, falls, road traffic collisions, accidental poisoning, fire/burns, animal related injuries (bite, sting, crush or attack).

Operational definition:
Home injury - Any injury defined above that occurs in the home environment but not related to work. (Work is any means by which someone earns money. So, if someone works from home, e.g., making something to sell, growing food / animals to sell – that is work and that would be an occupational injury). If you farm to grow food just for home/family use, that could be a home injury.

Occupational injury - Any injury defined above that occurs during paid employment at any location, including those who might be working without a wage but works for the benefit of their family (e.g. agriculture and animal husbandry).

DISCUSSION/INTERVIEW
Now we shall begin our discussion in an organized way. For this please think about the injury that occurs in the home environment/work environment to any age of people who live or work in your community. Please start with an example you know about. In addition, please give us some examples that you have seen in your home/working area.

[To organize the group discussion, use the following questions to guide the discussion]:

Part 1. Perceptions of injuries
1.1 What do we mean by injury?
1.2 What injuries have you experienced / witnessed in the home/work environment (as appropriate)

Part 2. Risk factors for injuries

Supplementary files

For peer review only - http://bmjopen.bmj.com/site/about/guidelines.xhtml

2.1 What caused those injuries in the home/work environment (as appropriate)? How did those injuries happen?

Part 3. Suggested intervention for injury prevention
3.1 What could be done (and by whom) to prevent other people having home/work injuries?
3.2 Home environment (as appropriate) - Whether the community people have had any change in their home environment and if so what that involved, and whether they think it helps? Also, do they have any safety equipment? If so, who provided it? Is it maintained? Does anyone check they are using it?
3.3 Work environment (as appropriate) - Whether the workers have had any safety training for their job and if so, what that involved and whether they think it helps? Also, do they have safety equipment? If so, who provides it? Is it maintained? Does anyone check they are using it?

Part 4. Barriers to and facilitators for such interventions
4.1 What might prevent such actions being done in the home/work environment (as appropriate)?
4.2 What might help such actions being done in the home/work environment (as appropriate)?

WRAP-UP

[At the end, there will be a short session for summarizing on a flip chart and recapping the whole discussion, thanking the participants and serving refreshments. This will be followed immediately by a meeting between the facilitator and note-taker]

Ending the discussion by saying: We have been discussing for about an hour. Do you think we missed anything that needs to be discussed?

FG discussion recording details

**Date:**
**Palika's name:**
**Ward No.:**
**Location (venue):**
**Type of Target Group:**
**Number of participants:**
**Age group:**
**Level of education:**
**Length of session:**
**Facilitator's name**
**Note taker' name:**
**Observation (if any):**
**Recorder No.:**

For peer review only - http://bmjopen.bmj.com/site/about/guidelines.xhtml

Supplementary file 2. A semi-structured interview schedule for key interviews

INTRODUCTION

Explain the study, ask if the participant has read the information sheet. Use the information sheet to explain the purpose of study, the voluntary nature of the interview, that the information they give will be kept confidential and that they can withdraw at any time.

Audio-recording: explain and ask for consent to use.

Consent: explain and ensure consent form is signed has a thumb print.

FOR REFERENCE IF DEFINITION NEEDED:

Injury definition: "The physical damage that results when a human body is subjected to energy that exceeds the threshold of physiological tolerance or results in lack of one or more vital elements, such as oxygen". All types of injury will be included in this study, including those described as intentional or unintentional. The terms intentional and unintentional denote whether an injury was meant to harm the victim or not. Intentional injuries include suicide and self-harm, homicide, assault and child abuse or purposeful neglect. Unintentional injury is any injury that originated suddenly, without any intent of self-harm, homicide or suicide. For example, falls, road traffic collisions, accidental poisoning, fire/burns, animal related injuries (bite, sting, crush or attack).

Operational definition:

Home injury: Any injury defined above that occur in the home environment (home and near environment) but not related to work. (Work is any means by which someone earns money. So, if someone works from home, e.g., making something to sell, growing food / animals to sell – that is work and that would be an occupational injury). If you farm to grow food just for home/family use, that could be a home injury.

Occupational injury: Any injury defined above that occurs during work at any location, including those who might be working without a wage but works for the benefit of their family (e.g. agriculture and animal husbandry).

Opening:

We would like to ask you a few questions about injuries, how they happen and what might prevent them. There are no right or wrong answers; we just want to find out what you think helps to keep people safe from accidents and what makes it hard to do this.

1. What is your understanding of what an injury is? Can you give an example, please?
   (if they are not correct, explain what we mean by the word 'injury')

2. What injuries have you experienced / witnessed in the home/work environment (as appropriate)? Can you describe one instance?

   (If they can't give an example, ask them to imagine a near miss or something that they could envisage happening.)

3. What do you think might have caused those injuries/why do you think they happened? How did those injuries happen? Can you describe the reasons, please?

   (Try and get them to give as much detail as possible. Repeat what they have said and ask them more details, if need be.)

4. Do you have any ideas about how injuries in the home/at work (as appropriate) could be prevented?
   a. What sort of thing might help?
   b. Who might do this?
   c. How might this be done?

For peer review only - http://bmjopen.bmj.com/site/about/guidelines.xhtml

5. Have you made any changes to your home/work to try and reduce the likelihood of an injury happening? Have you tried to make some changes?

    a. Has anyone in the community (home or work) made any changes? If yes, what? If yes, do you think it helped?
    b. Is any safety equipment used in the home/work at all? If yes, what and who provided it? Is it maintained (if relevant)? Does anyone check it (if relevant)?

(They might not be in a position to make any changes so ask whether they have ideas for what could have been done and if they have made suggestions to anyone.)

6. **Work environment:** have the workers had any safety training for their job, this includes any training for use of safety equipment? Who provided it? Is this undertaken regularly? Who ensures that it is completed?

Barriers and facilitators to injury prevention

7. What might prevent or deter people trying to use an intervention to stop injuries (home or work)? Why might people not use something that will help to prevent an injury? Do you have any ideas?

(Try to get some detail here about cost, practicality, someone taking ownership, leading the intervention etc.)

8. What might help or encourage people to use an intervention to stop injuries (home or work)? Do you have any ideas?

(Try to get some detail here about cost, practicality, someone taking ownership, leading the intervention etc.)

9. Do you have any other comments or anything else that you would like to say?

Thank you very much for talking to me. We will use the information that you have given me to describe the hazards that cause injuries and the possible interventions that might be used to help prevent them.

KIIs recording details

**Date:**
**Palika's name:**
**Ward No.:**
**Location (venue):**
**Type of Target Group:**
**Age group:**
**Level of education:**
**Length of interview:**
**Interviewer's name:**
**Observation (if any):**
**Recorder No.:**
**Personal identification code**

Supplementary files

For peer review only - http://bmjopen.bmj.com/site/about/guidelines.xhtml

Perceptions of injury risk in the home and workplace in Nepal: a qualitative study

Consolidated criteria for reporting qualitative studies (COREQ): 32-item checklist (Tong, Sainsbury & Craig, 2007)

No. Item	Guide questions	Description/Page
**Domain 1: Research team and reflexivity**		
Personal Characteristics		
1. Interviewer/facilitator	Which author/s conducted the interview or focus group?	EJ facilitated 7 Focus groups and 8 interviews and SB facilitated 2 focus groups and 1 interview Page: 5, DA helped to organize focus groups and interviews
2. Credentials	What were the researcher's credentials? E.g. PhD, MD	EJ: Masters in Nursing<br>SB: PhD<br>TD: PhD<br>JM: PhD<br>SRM: MD<br>DA: Masters in Social Science<br>SKJ: PhD
3. Occupation	What was their occupation at the time of the study?	TD, EJ, SB, JM: Researchers<br>DA: Site Coordinator<br>SKJ: Researcher, Academician<br>SRM: Researcher, Pediatrician
4. Gender	Was the researcher male or female?	TD, EJ. JM: Female<br>SB, SRM, SKJ, DA: Male
5. Experience and training	What experience or training did the researcher have?	All authors have relevant research experience and training on study design, data collection and analysis
Relationship with participants		
6. Relationship established	Was a relationship established prior to study commencement?	No relationship was established prior to study.
7. Participant knowledge of the interviewer	What did the participants know about the researcher? e.g. personal goals, reasons for doing the research	The participants were briefed about research objectives using study information sheet. Page:4
8. Interviewer characteristics	What characteristics were reported about the inter viewer/facilitator? e.g. Bias, assumptions, reasons and interests in the research topic	Trained researchers with experience in conducting injury research undertook the data collection.
**Domain 2: study design**		
Theoretical framework		
9. Methodological orientation and Theory	What methodological orientation was stated to underpin the study? e.g. grounded theory, discourse analysis, ethnography, phenomenology, content analysis	Inductive thematic analysis was done and the rationale for using it has been justified in the method section. Page:5
Participant selection		
10. Sampling	How were participants selected? e.g. purposive, convenience, consecutive, snowball	Purposive sampling using pre-specified sampling framework. Page:4-5

For peer review only

Perceptions of injury risk in the home and workplace in Nepal: a qualitative study

Consolidated criteria for reporting qualitative studies (COREQ): 32-item checklist (Tong, Sainsbury & Craig, 2007)

11. Method of approach	How were participants approached? e.g. face-to-face, telephone, mail, email	Face to face interviews and focus groups were conducted. Page:3-4
12. Sample size	How many participants were in the study?	A total of 83 in nine focus groups and nine interviews in eighteen different sessions. Page:5
13. Non-participation	How many people refused to participate or dropped out? Reasons?	One participant refused to participate. No reason was given.
Setting		
14. Setting of data collection	Where was the data collected? e.g. home, clinic, workplace	The data were collected at home and workplace, whichever was convenient and feasible for the participants. Page: 4
15. Presence of non-participants	Was anyone else present besides the participants and researchers?	A local field staff took notes during the focus group discussions. The field staff were not familiar with the participants.
16. Description of sample	What are the important characteristics of the sample? e.g. demographic data, date	Sex, age, participants role, location Page:6-7
Data collection		
17. Interview guide	Were questions, prompts, guides provided by the authors? Was it pilot tested?	Topic guide and interview schedule were developed for focus groups and interviews, respectively. The topic guide and interview schedule were pre-tested. Page:5
18. Repeat interviews	Were repeat inter views carried out? If yes, how many?	No.
19. Audio/visual recording	Did the research use audio or visual recording to collect the data?	Audio recording of focus groups and interviews with participants consent Page:4-5
20. Field notes	Were field notes made during and/or after the interview or focus group?	Brief field and observation notes were made during focus groups.
21. Duration	What was the duration of the inter views or focus group?	The average focus groups were 37 minutes and interviews were 31 minutes. Page:6
22. Data saturation	Was data saturation discussed?	No. Not required as per the study aim. Page: 4
23. Transcripts returned	Were transcripts returned to participants for comment and/or correction?	The transcripts were not returned to participants.
**Domain 3: analysis and findings**		
Data analysis		
24. Number of data coders	How many data coders coded the data?	Two
25. Description of the coding tree	Did authors provide a description of the coding tree?	No. Can be made available if requested
26. Derivation of themes	Were themes identified in advance or	Identified from the data.

For peer review only

Perceptions of injury risk in the home and workplace in Nepal: a qualitative study

Consolidated criteria for reporting qualitative studies (COREQ): 32-item checklist (Tong, Sainsbury & Craig, 2007)

	derived from the data?	
27. Software	What software, if applicable, was used to manage the data?	Translation, transcription, and data analysis were done manually with the use of NVivo to manage the data.
28. Participant checking	Did participants provide feedback on the findings?	The participants didn't provide feedback on the findings.
Reporting		
29. Quotations presented	Were participant quotations presented to illustrate the themes/findings? Was each quotation identified? e.g. participant number	The quotes from participants were used and identified using a personal identification code to ensure anonymity. Page:7-11
30. Data and findings consistent	Was there consistency between the data presented and the findings?	There was consistency between the data presented and the findings.
31. Clarity of major themes	Were major themes clearly presented in the findings?	Yes. Major themes are described. Page: 7-11
32. Clarity of minor themes	Is there a description of diverse cases or discussion of minor themes?	We presented the major themes. Diversity of the participants are described. Page: 4 & 5

For peer review only
